# Exon junction complex and nuclear pore complex components preferentially govern the nuclear export of spliced lncRNAs

**DOI:** 10.1038/s41420-026-03246-0

**Published:** 2026-07-08

**Authors:** Sondas Saeed, Weijie Wang, Chaoqun Chen, Xiaokun Yuan, Siqi Zhao, Xin Xu, Xinyi Xu, Jiaxin Liu, Shuai Hou, Jinsong Yan, Haixin Lei

**Affiliations:** 1 Institute of Cancer Stem Cell, Dalian Medical University, Dalian, China; 2 Liaoning Provincial Key Laboratory of Nucleic Acids Biology, Dalian Medical University, Dalian, China; 3 International Peace Maternity and Child Health Hospital, School of Medicine, Shanghai Jiao Tong University, Shanghai, China; 4 Department of Hematology, Liaoning Medical Center for Hematopoietic Stem Cell Transplantation, The Second Hospital of Dalian Medical University, Dalian, China; 5 School of Basic Medical Sciences, Hainan Medical University, Haikou, China

**Keywords:** Long non-coding RNAs, RNA transport

## Abstract

Long non-coding RNAs (lncRNAs) are crucial regulators of gene expression, but how spliced lncRNAs destined for the cytoplasm are exported from the nucleus remains unclear. Using LINC00998 and ANCR as cytoplasmic lncRNA reporters, in vivo-assembled lncRNPs were purified via MS2-MBP-based affinity purification, and proteins in the lncRNPs were identified by mass spectrometry. An siRNA screen followed by RNA FISH showed that while knockdown of the TREX-TAP pathway components UAP56 and TAP blocked the export of both lncRNAs and mRNAs, depletion of ALYREF, the exon junction complex (EJC) core protein eIF4A3, or the nuclear pore complex (NPC) component NUP98 preferentially impaired lncRNA export without significantly affecting spliced mRNAs. This lncRNA-biased retention phenotype was also observed upon depletion of other EJC components, Y14 or MAGOH/MAGOHB, and at least 9 additional NPC components. In contrast, depletion of NUP160 or NUP205 caused strong retention of spliced lncRNAs and partial retention of spliced mRNAs. Consistent with these findings, stable knockdown of eIF4A3 and NUP98 predominantly increased nuclear accumulation of endogenous lncRNAs, whereas NUP160 depletion affected both RNA classes. Overall, our results demonstrate that although spliced lncRNAs utilize the canonical mRNA export machinery, they exhibit a distinct and preferential dependency on specific EJC and NPC components.

**Figure:**
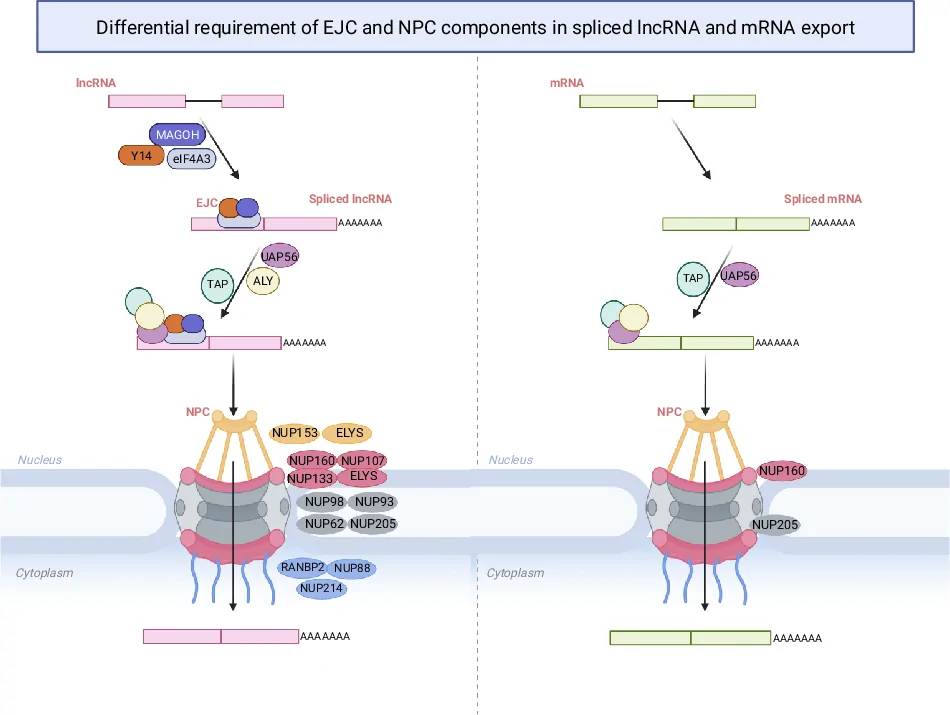

## Introduction

Gene expression in eukaryotic cells depends on the coordinated regulation of transcription, RNA processing, export, translation, and decay. Among these steps, the export of RNA from the nucleus to the cytoplasm is an important checkpoint that coordinates nuclear RNA maturation with gene expression in the cytoplasm.

Long non-coding RNAs (lncRNAs) are RNA molecules more than 200 nucleotides in length. They lack protein-coding function but play an important role in cellular processes, including chromatin remodeling, transcription, and RNA processing [1]. Many lncRNAs are transcribed from intergenic regions and go through the same processing steps as mRNAs, like capping, splicing, and polyadenylation [2]. However, unlike mRNAs, which are universally exported to the cytoplasm for translation, lncRNAs may localize in the nucleus, cytoplasm, or in both compartments [3, 4]. The subcellular localization of lncRNAs is fundamental to their function, as it determines their modes of action. Nuclear lncRNAs typically function in transcriptional regulation and chromatin modification [5–7], while cytoplasmic lncRNAs participate in post-transcriptional control and signaling pathways [8, 9]. However, for spliced lncRNAs localized in the cytoplasm, little is known about how they are exported.

Recent studies have revealed complex mechanisms governing lncRNA nuclear retention and cytoplasmic export. A subset of lncRNAs has distinct cis-acting motifs that distinguish them from cytoplasmic RNAs, resulting in nuclear retention [10, 11]. These include nuclear retention elements (NREs) that recruit U1 snRNP components, C-rich motifs that interact with hnRNPs, and 5’ splice site-like sequences that promote nuclear retention [12–14]. Trans-acting factors, including RNA helicases and RNA processing factors, coordinately interact with these motifs to restrict lncRNAs in the nucleus [10]. However, inefficient splicing, which is more common in lncRNAs than mRNAs, often leads to preferred nuclear localization [15]. Conversely, cytoplasmic lncRNAs must successfully recruit export machinery during their maturation in the nucleus to overcome these retention mechanisms. Studies on intronless lncRNAs like NKILA have demonstrated that sequence-dependent recruitment of SR proteins SRSF1/SRSF7 facilitates TREX complex assembly and nuclear export via the TREX-TAP pathway [16]. Moreover, proteins like TPR in the nuclear basket are necessary for efficiently exporting lncRNAs and mRNAs from short, intron-poor genes [17]. In addition, the sequence and structure of lncRNAs also play a role in their export or retention, showing a complex regulatory scheme [18]. These findings emphasize that lncRNA export cannot be fully explained by traditional mRNA pathways but instead involves unique regulatory features and mechanisms that distinguish them from protein-coding transcripts.

For mRNAs, the export pathway usually involves the TREX complex and the nuclear pore complex (NPC). Splicing-dependent TREX recruitment represents a major pathway for mRNA export [19]. Structural studies show that TREX creates a hub for protein–RNA interactions, helping stabilize RNA-protein complexes (RNP) and prepare them for transport [20, 21]. Recent studies have also revealed that mRNA export is influenced by RNA modifications, particularly N6-methyladenosine (m6A), which coordinates with the TREX complex to facilitate export [22, 23]. Furthermore, competition between export adaptors and nuclear RNA surveillance machinery determines whether polyadenylated RNAs are exported or degraded, highlighting the dynamic regulation of mRNA nuclear-cytoplasmic trafficking [24].

All the matured mRNAs need to pass the NPC in order to complete the process of RNA export. The NPC is a large protein structure that actively controls selective exchange between the nucleus and cytoplasm, rather than serving as a passive channel. Composed of multiple nucleoporins (NUPs), it serves as a structural framework and dynamic interaction site for export adaptors and receptors [25, 26]. For instance, NUP98 plays a critical role in mRNA export by interacting with the export receptor TAP and has been linked to multiple RNA export steps [27, 28]. NUP160, along with its associated proteins in the Nup107-160 subcomplex, is crucial for NPC assembly and nucleocytoplasmic transport [29, 30]. Beyond mRNA and lncRNA export, TPR also coordinates the nuclear export of tRNAs and rRNAs in association with TAP, a function found to be particularly critical in lung cancer cells [31]. Studies using systematic deletion of FG domains in yeast revealed that specific nucleoporins NUP57 and NUP49 are preferentially required for mRNA export [32]. These findings suggest that individual nucleoporins can favor the export of specific RNA classes.

In this study, we aimed to identify nuclear factors that regulate the export of spliced lncRNAs and to determine whether these factors are distinct from those controlling mRNA export. Our findings revealed that efficient spliced lncRNA export depends on the exon junction complex (EJC) core component eIF4A3 and its associated factors, selective engagement of the TREX adaptor ALYREF, and passage through the nuclear pore via specific nucleoporins, most notably NUP98. This multi-layered EJC-ALYREF-NPC axis distinguishes spliced lncRNA export from canonical mRNA.

## Results

### Establishment of lncRNA export reporter systems and identification of associated RNA-binding proteins

**Fig. 1:**
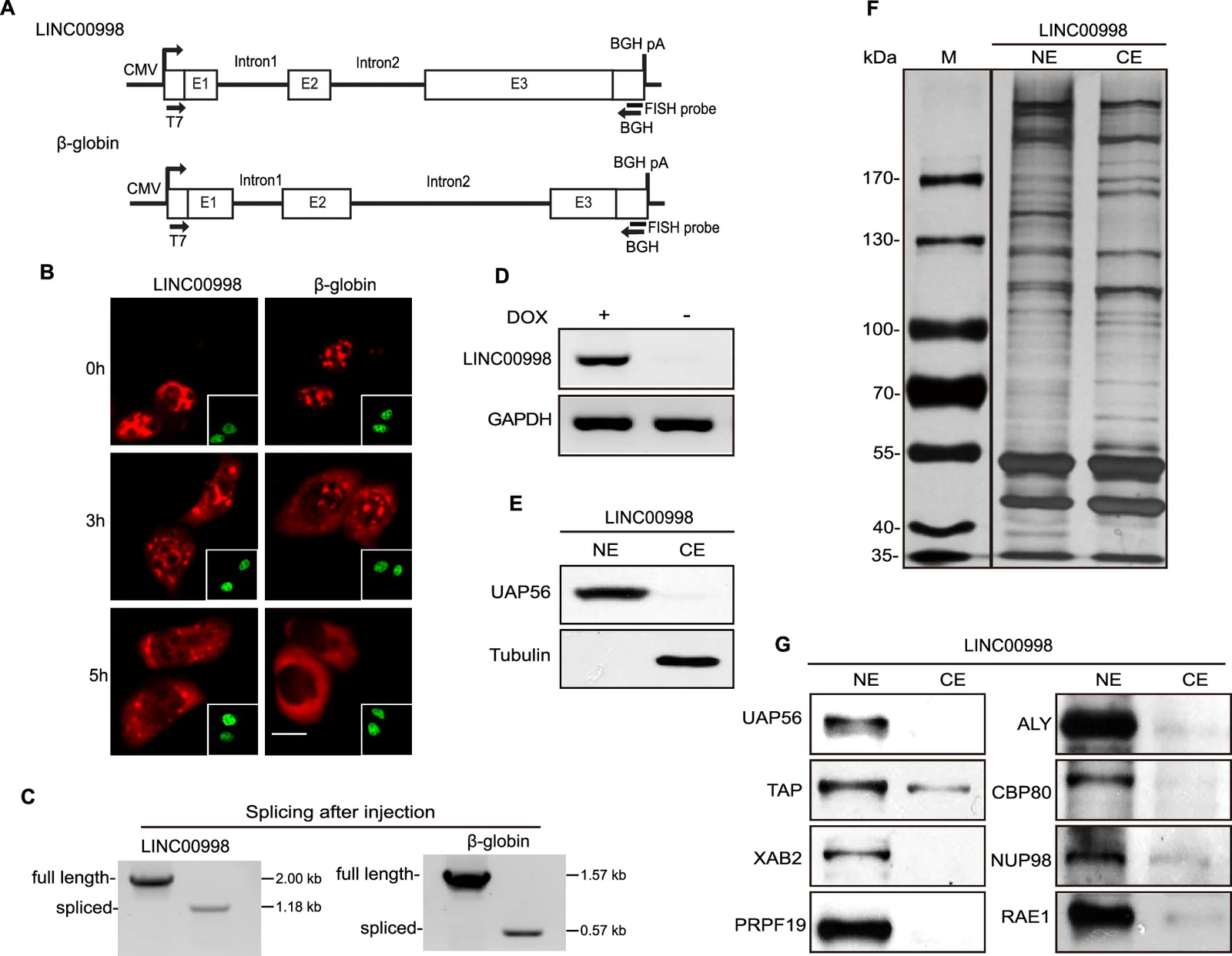
Subcellular localization and lncRNPs purification of spliced lncRNA reporter LINC00998. **A** Schematic representation of the reporter constructs for LINC00998 and β-globin in pcDNA 3.1, each driven by the CMV promoter, having BGH poly(A) signal. White boxes represent exons, and black lines indicate introns. T7 and BGH indicate plasmid-specific primers. **B** RNA-FISH analysis of nuclear and cytoplasmic distribution of reporter transcripts at the indicated time points following nuclear microinjection into HeLa cells. Transcription was blocked with α-amanitin 30 min after injection (defined as 0 h). Scale bar = 5 µm. **C** RT-PCR analysis using T7 and BGH primers, confirming normal splicing of LINC00998, and β-globin reporter transcripts following microinjection. **D** Establishment of DOX-inducible FRT-HeLa cell lines stably expressing LINC00998. Expression was induced by DOX treatment and detected by RT-PCR using LINC00998-KpnI-F and LINC00998-BamHI-R primers. GAPDH is used as the loading control. **E** Validation of nuclear and cytoplasmic fractionation by western blot using UAP56 and tubulin as nuclear and cytoplasmic markers, respectively. **F** Silver staining of purified lncRNPs from nuclear and cytoplasmic fractions showing distinct banding patterns. **G** Western blot analysis confirming the interaction of selected proteins identified by mass spectrometry with LINC00998.

To investigate the factors important for spliced lncRNA export, we first constructed two lncRNA reporters with the transcripts reported to be localized in the cytoplasm. Both reporters contained full genomic sequences covering LINC00998 and ANCR, and a β-globin wild-type reporter was used as a spliced mRNA control (Fig. 1A and Suppl Fig. 1A). All the reporters were driven by the CMV promoter and contained the BGH poly(A) signal. These reporter plasmids were then microinjected into the nuclei of HeLa cells. RNA-FISH was used to detect the nuclear and cytoplasmic distribution of the reporter transcripts at various time points. Thirty minutes after microinjection, α-amanitin was added to block transcription, which marked the 0 h time point; at this time, all reporter transcripts were localized in the nucleus (Fig. 1B and Suppl Fig. 1B). After 3 h, most β-globin transcripts were transported to the cytoplasm, while most of the lncRNA remained in the nucleus, with only a small portion detected in the cytoplasm. By 5 h, most lncRNA reporter transcripts were also transported to the cytoplasm (Fig. 1B and Suppl Fig. 1B). RT-PCR confirmed that all reporter transcripts underwent normal splicing after microinjection (Fig. 1C and Suppl Fig. 1C). These data together suggest that both lncRNAs can be exported to the cytoplasm, but the kinetics seems to be not as fast as spliced β-globin mRNA.

Further, to identify proteins crucial for the export of these lncRNAs, we established doxycycline (DOX)-inducible cell lines stably expressing each lncRNA and purified lncRNPs assembled both in the nucleus and the cytoplasm using an MS2-MBP based strategy. As shown in Fig. 1D and Suppl Fig. 1D, induction of DOX led to the detection of the expression of specified lncRNA. After induction, nuclear and cytoplasmic fractions were separated, and the fractionation efficiency was verified by western blot, using UAP56 and tubulin as nuclear and cytoplasmic markers, respectively (Fig. 1E and Suppl Fig. 1E). RNPs purified from the nucleus or cytoplasm were then separated on polyacrylamide gel followed by silver staining, which showed a distinct pattern (Fig. 1F and Suppl Fig. 1F). The components in the RNPs were identified by mass spectrometry and shown in Suppl Table 1. Several proteins identified in the nuclear RNPs were verified using western blot as shown in Fig. 1G and Suppl Fig. 1G. Collectively, these data confirm the successful establishment of spliced lncRNA reporter models and uncover candidate proteins involved in their nuclear export.

### UAP56, TAP, and ALYREF mediate nuclear export of lncRNAs

**Fig. 2:**
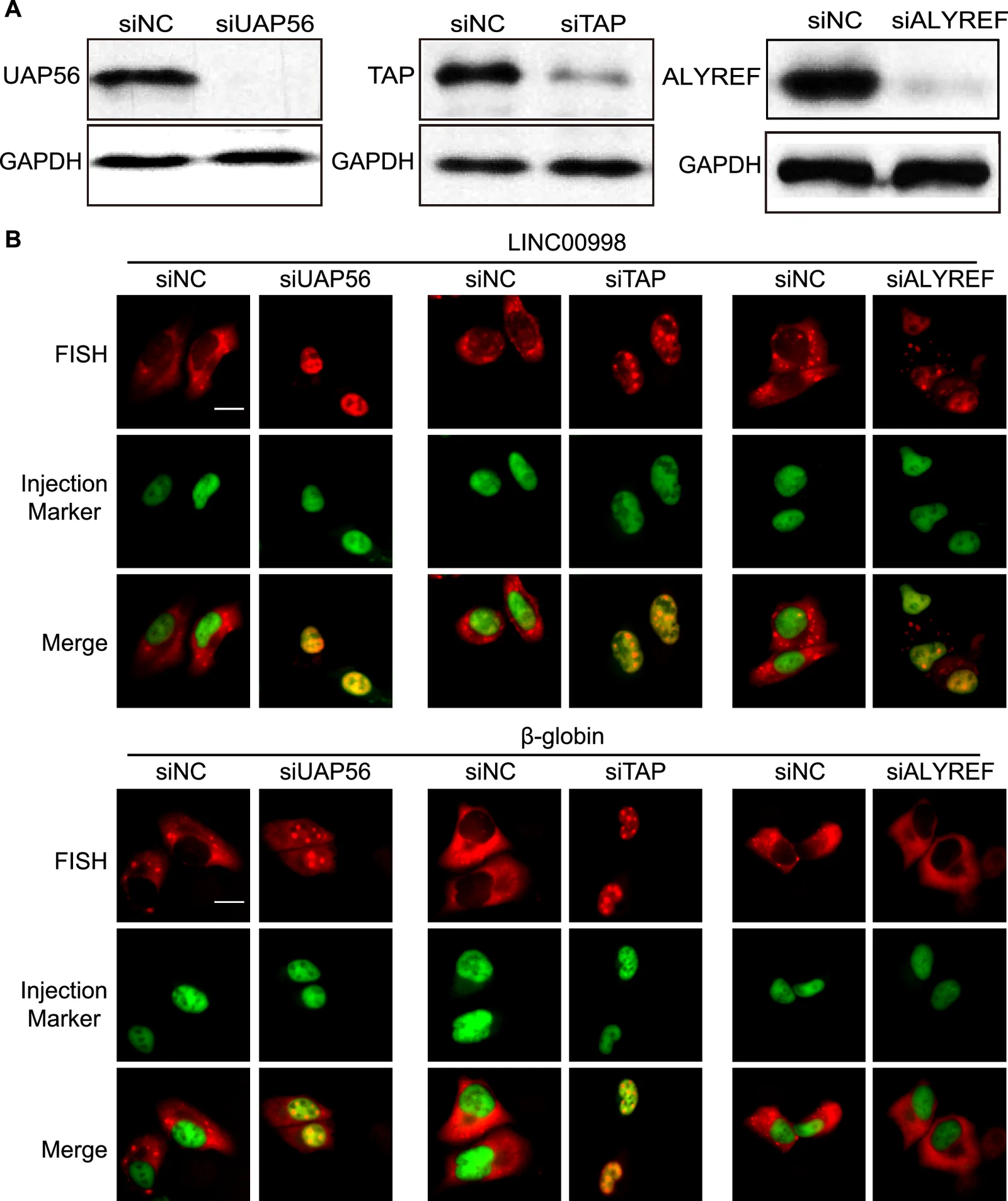
TREX-TAP components mediate nuclear export of lncRNAs and mRNAs. **A** Western blot validation of siRNA-mediated knockdown of UAP56, TAP, and ALYREF in HeLa cells. GAPDH serves as a loading control. **B** RNA-FISH analysis of nuclear and cytoplasmic localization of LINC00998 and β-globin reporter transcripts post-microinjection following knockdown of UAP56, TAP, or ALYREF. Scale bar = 5 µm.

To determine whether proteins identified by mass spectrometry contribute to lncRNA export, we first focused on DDX39B (UAP56) and ALYREF, core components of the TREX-TAP pathway critical for mRNA export. Since NXF1 (TAP) is the canonical downstream export receptor that receives RNA cargo from UAP56 and ALYREF in this pathway, we also examined its role, extending our analysis beyond the mass spectrometry data to cover the full TREX-TAP export axis. siRNA-mediated knockdown of each protein was validated by western blot (Fig. 2A). The reporters were then microinjected into the nuclei of knockdown cells to examine the effects on nuclear export. As observed in Fig. 2B and Suppl Fig. 2, depletion of UAP56 or TAP not only blocked the export of spliced mRNA as reported previously, but it also caused severe export defects for lncRNAs. In contrast, depletion of ALYREF selectively impaired lncRNA export, while β-globin mRNA export remained largely unaffected (Fig. 2B and Suppl Fig. 2). Together, these results demonstrate that TREX-TAP pathway is critical for the export of both mRNA and lncRNA, but lncRNAs exhibit a specific dependence on ALYREF, highlighting mechanistic differences between their export pathways.

### Identification of EJC component eIF4A3 as a critical factor for lncRNA export

**Fig. 3:**
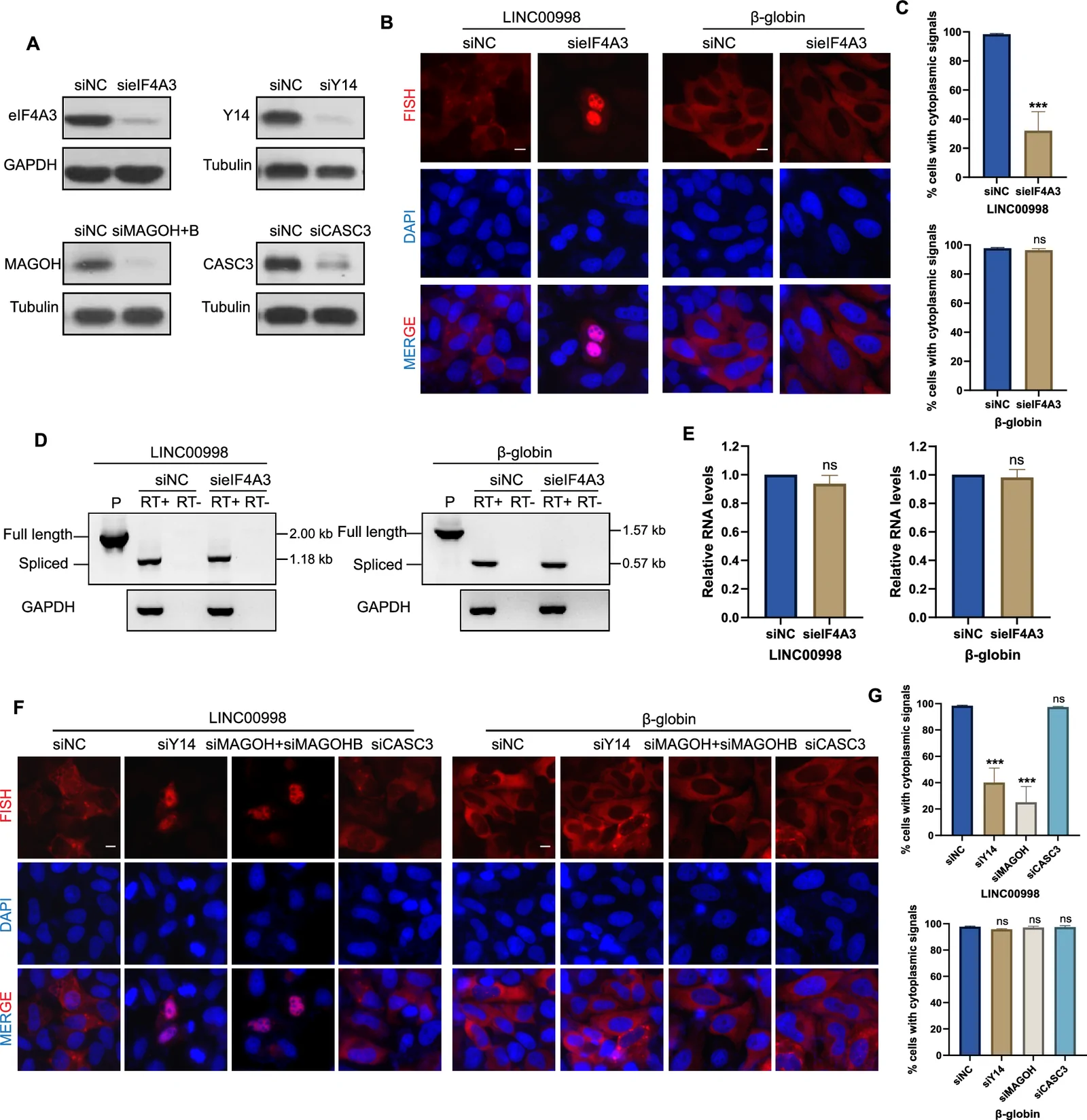
eIF4A3 and other core EJC components preferentially mediate lncRNA nuclear export. **A** Western blot validation of siRNA-mediated knockdown of eIF4A3, Y14, MAGOH/MAGOHB, and CASC3 in HeLa cells. **B** RNA-FISH analysis of subcellular localization of LINC00998 and β-globin reporter transcripts post-transfection following eIF4A3 knockdown. Scale bar = 10 µm. **C** Quantification of cells with predominant cytoplasmic signals for LINC00998 and β-globin following eIF4A3 knockdown (*n* = 3, ns: not significant, ***: *p* < 0.001). **D** RT-PCR analysis of splicing and overall abundance of LINC00998 and β-globin reporter transcripts following eIF4A3 knockdown using T7 and BGH primers. P: plasmid control; RT + : reverse transcriptase added; RT-: no reverse transcriptase control. **E** Quantification of relative RNA levels of LINC00998 and β-globin following eIF4A3 knockdown showing no significant change in RNA abundance (*n* = 3, ns: not significant). **F** RNA-FISH analysis of subcellular localization of LINC00998 and β-globin reporter transcripts following knockdown of Y14, MAGOH/MAGOHB, and CASC3. Scale bar = 10 µm. **G** Quantification of cells with predominant cytoplasmic signals for LINC00998 and β-globin following knockdown of individual EJC components (*n* = 3, ns: not significant, ***: *p* < 0.001).

To further identify trans-factors that may function in lncRNA export, we next screened additional proteins detected in the mass spectrometry dataset based on their known roles in RNA biogenesis, nuclear RNA metabolism, and export pathways. Screening of these proteins using siRNA, followed by RNA-FISH for RNA localization, revealed that eIF4A3, a core component of EJC, played a vital role in the export of spliced lncRNA. The knockdown efficiency of eIF4A3 was confirmed using western blot (Fig. 3A). Depletion of eIF4A3 resulted in significant nuclear retention of spliced LINC00998 and ANCR, whereas localization of spliced β-globin mRNA was not significantly altered (Fig. 3B, C and Suppl Fig. 3A, B). This observation was further verified using another spliced mRNA reporter, GADD45, and, similarly to β-globin, no significant export defect was observed for transcripts from this construct (Suppl Fig. 3C–E).

Importantly, RT-PCR analysis of reporter transcripts following depletion of eIF4A3 revealed that all reporter transcripts underwent normal splicing, and the abundance of spliced RNAs was not detectably altered, indicating that the observed nuclear accumulation is unlikely to result from defects in RNA splicing or stability (Fig. 3D, E and Suppl Fig. 3F, G). Consistently, a second independent siRNA targeting eIF4A3 produced the same nuclear retention phenotype for spliced LINC00998 and ANCR, confirming that the observed effects are specific to eIF4A3 depletion (Suppl Fig. 4A–C).

As eIF4A3 is a core component of EJC, we next investigated whether other components of this complex were also involved in lncRNA export. We depleted core EJC components, including Y14, MAGOH/MAGOHB, and CASC3 (Fig. 3A), and examined the localization of transcripts from different reporters. Strikingly, depletion of Y14 and MAGOH/MAGOHB resulted in significant nuclear retention of lncRNA transcripts, whereas depletion of CASC3 did not have any apparent effect (Fig. 3F, G and Suppl Fig. 3H, I). Importantly, depletion of these factors did not significantly affect the export of spliced β-globin and GADD45 mRNAs (Fig. 3F, G and Suppl Fig. 3H, I), suggesting that the dependence on EJC components for nuclear export is lncRNA-biased. Together, these results identify eIF4A3 and the associated EJC components Y14 and MAGOH/MAGOHB as essential mediators of spliced lncRNA export.

### NUP98 and other NPC components differentially contribute to lncRNA export

**Fig. 4:**
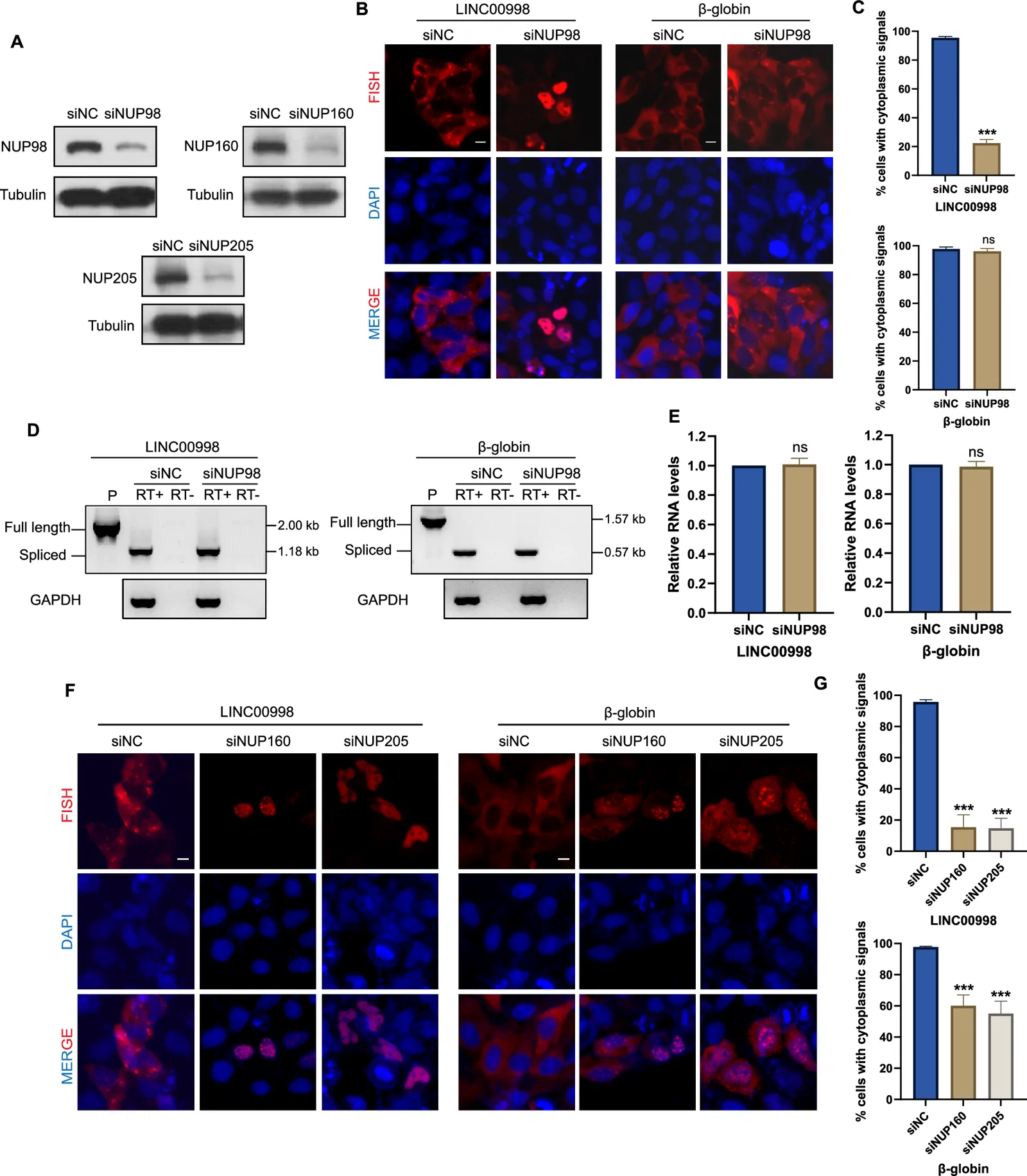
NUP98 and specific NUPs differentially regulate lncRNA nuclear export. **A** Western blot validation of siRNA-mediated knockdown of NUP98, NUP160, and NUP205 in HeLa cells. **B** Subcellular localization of LINC00998 and β-globin reporter transcripts post-transfection following NUP98 knockdown. Scale bar = 10 µm. **C** Quantification of cells with predominant cytoplasmic signals for LINC00998 and β-globin following NUP98 knockdown (*n* = 3, ns: not significant, ***: *p* < 0.001). **D** RT-PCR analysis of splicing and overall abundance of LINC00998 and β-globin reporter transcripts following NUP98 knockdown using T7 and BGH primers. P: plasmid control; RT+: reverse transcriptase added; RT-: no reverse transcriptase control. **E** Quantification of relative RNA levels of LINC00998 and β-globin following NUP98 knockdown, showing no significant change in RNA abundance (*n* = 3, ns: not significant). **F** RNA-FISH analysis of subcellular localization of LINC00998 and β-globin reporter transcripts following knockdown of NUP160 and NUP205. Scale bar = 10 µm. **G** Quantification of cells with predominant cytoplasmic signals for LINC00998 and β-globin following knockdown of NUP160 and NUP205 (*n* = 3, ***: *p* < 0.001).

Given that NUP98 was also detected in our mass spectrometry dataset and represents a key NPC component, we next examined its role in lncRNA export alongside other nucleoporins (NUPs). siRNA-mediated depletion of NUP98 in HeLa cells was confirmed by western blot (Fig. 4A), and RNA-FISH revealed that knockdown of NUP98 caused strong nuclear retention of both LINC00998 and ANCR, whereas no significant effect on localization of spliced β-globin and GADD45 mRNAs was observed (Fig. 4B, C and Suppl Fig. 5A, B).

RT-PCR analysis of reporter transcripts following depletion of NUP98 also revealed that all reporter transcripts underwent normal splicing, and the abundance of spliced RNAs was not detectably altered, indicating that the observed nuclear accumulation is unlikely to result from defects in RNA splicing or stability (Fig. 4D, E and Suppl Fig. 5C, D). The effect of NUP98 was further confirmed using a second independent siRNA, which showed the same nuclear retention phenotype for spliced LINC00998 and ANCR with no significant effect on spliced mRNA reporters, further supporting the specificity of the observed phenotype (Suppl Fig. 6A–C).

To assess the impact of other NPC components, we knocked down a panel of NUPs using siRNAs (Fig. 4A and Suppl Fig. 7A). NUP160 or NUP205 knockdown resulted in significant nuclear retention of both lncRNAs (LINC00998 and ANCR) as well as partial retention of β-globin and GADD45 mRNAs, indicating that these nucleoporins contribute to the export of both lncRNAs and mRNAs (Fig. 4F, G and Suppl Fig. 5E, F). In addition, depletion of other NUPs, including NUP62, NUP93, NUP133, RanBP2, NUP214, NUP88, NUP153, NUP107, and ELYS, caused nuclear retention of lncRNAs, with no apparent effect on mRNA export (Suppl Fig. 7B–E). These results indicate that the NPC components differentially affect the export of spliced lncRNAs and mRNAs, while NUP160 or NUP205 depletion impairs the export of both RNA types, depletion of a broader set of NUPs appears to specifically impairs lncRNA export.

### Preferential retention of endogenous lncRNAs upon eIF4A3 or NUP98 depletion versus general export defects for both lncRNAs and mRNAs upon NUP160 depletion

**Fig. 5:**
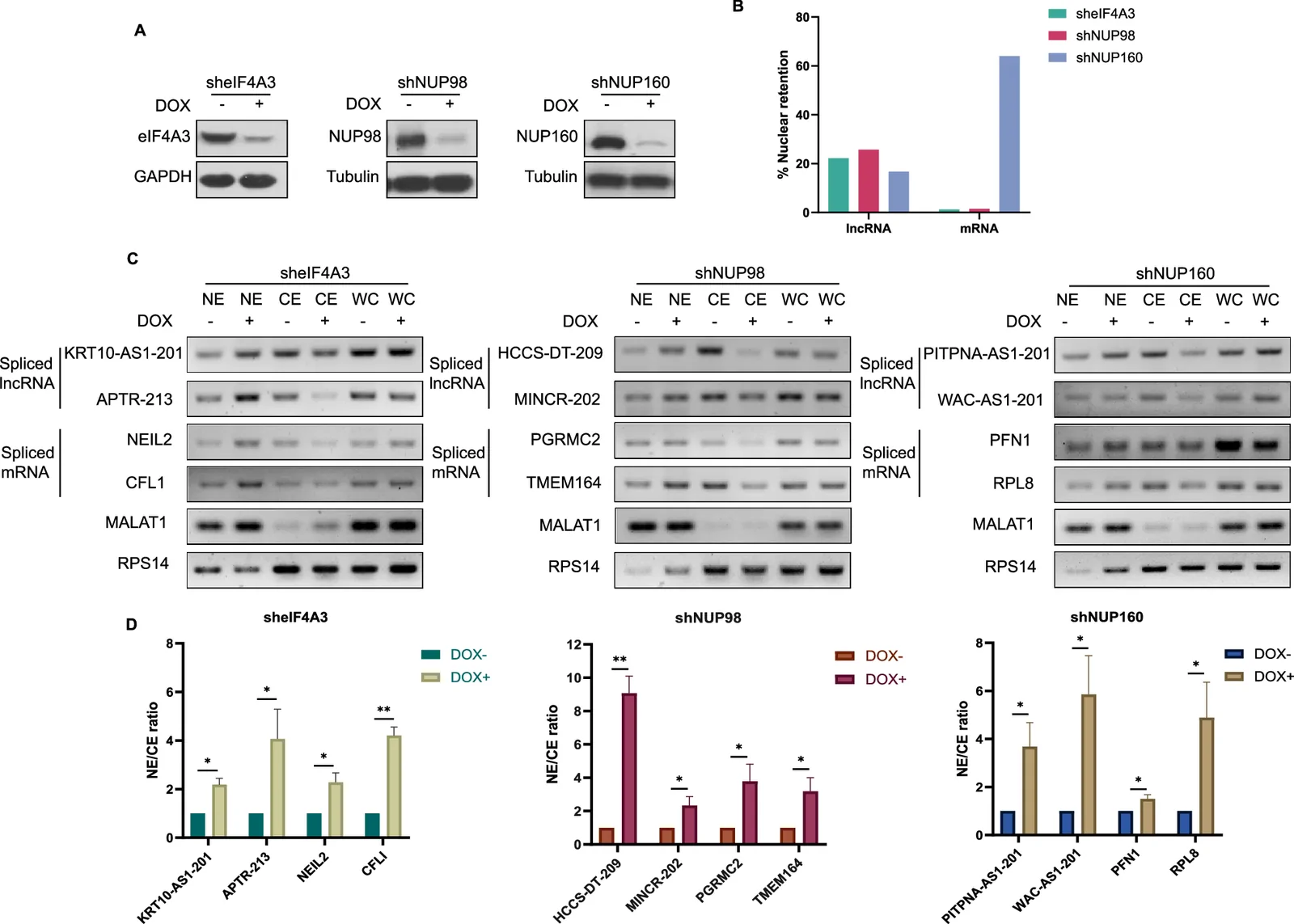
Knockdown of eIF4A3, NUP98, and NUP160 leads to nuclear retention of endogenous lncRNAs. **A** Western blot confirming efficient depletion of eIF4A3, NUP98, and NUP160 following DOX treatment. **B** Percentage of cytoplasmic polyadenylated spliced lncRNA transcripts and mRNA genes that are retained in the nucleus upon DOX-inducible knockdown. **C** RT-PCR using primers that specifically detect spliced transcripts showing the distribution of endogenous RNAs in respective stable knockdown cell lines. CE cytoplasmic fraction; NE nuclear fraction; WC whole cell. **D** Quantification of RT-PCR results (*n* = 3, *p < 0.05, **p < 0.01).

To further clarify the impact of these factors (eIF4A3, NUP98, NUP160) on the export of endogenous lncRNAs, we established DOX-inducible stable knockdown cell lines. Efficient depletion of each target was confirmed following DOX treatment (Fig. 5A). RNA-FISH analysis in these stable cell lines reproduced the reporter phenotypes observed with transient siRNA knockdown: spliced lncRNAs transcribed from reporters were retained in the nucleus upon eIF4A3, NUP98, and NUP160 depletion, whereas only NUP160 knockdown caused partial nuclear retention of spliced mRNAs transcribed from reporters (Suppl Fig. 8A–C).

To assess transcriptome-wide consequences, we separated nuclear and cytoplasmic fractions, performed RNA sequencing, and analyzed the subcellular localization patterns of spliced cytoplasmic polyadenylated transcripts by calculating the nuclear retention score (NRS). Transcripts with NRS > 1 were considered as nuclear retained after knockdown. In eIF4A3 knockdown cells, of 3706 lncRNA transcripts analyzed, 823 (22.21%) showed nuclear retention, whereas only 40 (1.25%) of 3,211 mRNA genes were nuclear-enriched. Similarly, in NUP98 knockdown cells, 920 (25.71%) of 3579 lncRNA transcripts were nuclear retained, compared to only 36 (1.50%) of 2393 mRNA genes. In striking contrast, NUP160 knockdown showed a markedly different pattern: 2742 (16.68%) of 16,439 lncRNA transcripts were nuclear-enriched, alongside 3745 (64.05%) of 5847 mRNA genes, demonstrating broad impairment of both RNA classes. (Fig. 5B and Suppl Table 2). To further confirm the sequencing results, we selected some nuclear-enriched spliced lncRNA transcripts and mRNA genes having sufficient expression levels, from these datasets and assessed their endogenous subcellular localization by RT-PCR, and this analysis confirmed the nuclear retention patterns as revealed by RNA-seq (Fig. 5C, D). Notably, while eIF4A3 and NUP98 depletion predominantly affected lncRNA nuclear export, a very small subset of mRNAs (referred to as the eIF4A3/NUP98-sensitive mRNA fraction) also exhibited nuclear retention, which was further confirmed by RT-PCR analysis (Fig. 5C, D). To rule out the possibility that splicing efficiency was affected after the knockdown of eIF4A3, apart from the splicing primers (EE) used to assess the endogenous expression, another set of primer (EI) was designed to amplify pre-spliced lncRNA. Apparently, no significant effect on both mature and spliced lncRNA cellular levels was observed under eIF4A3 knockdown conditions, indicating that splicing efficiency remains unchanged (Suppl Fig. 9). Taken together, these results demonstrate that eIF4A3 and NUP98 preferentially function in the nuclear export of spliced lncRNAs, whereas NUP160 is required for the export of both spliced lncRNAs and mRNAs.

To determine whether the nuclear retention observed upon eIF4A3, NUP98, or NUP160 knockdown reflects transcript architecture rather than RNA class identity, we examined correlations between nuclear enrichment and transcript features, including GC content, exon number, transcript length, and splice site strength (Suppl Figs. 10–13). Across eIF4A3 and NUP98 knockdown conditions, none of these features showed biologically meaningful correlations with lncRNA nuclear retention. In contrast, NUP160 knockdown, which impairs export of both RNA classes, revealed a strong positive correlation between GC content and nuclear retention for lncRNAs (Spearman *ρ* = 0.331, *p* = 0.000e + 00) and mRNAs (Spearman *ρ* = 0.753, *p* = 0.000e + 00) (Suppl Fig. 10E, F), suggesting that NUP160-dependent export operates through a GC content-driven mechanism shared across RNA classes. These findings indicate that the lncRNA-biased export dependency on eIF4A3 and NUP98 is unlikely to be explained by the transcript features examined.

## Discussion

Despite their emerging importance, the mechanisms governing nuclear export of spliced lncRNAs remain poorly understood. In this study, we established functional reporter systems for two spliced cytoplasmic lncRNAs, LINC00998 and ANCR, and systematically characterized the factors required for their export. We demonstrated that spliced lncRNAs engage canonical mRNA export machinery but exhibit preferential dependence on different EJC and NPC components.

To ensure that these reporters are representative of the broader lncRNA population, we compared their structural features against global lncRNA characteristics [33]. Both LINC00998 (1011 nt, 3 exons, 35.41% GC) and ANCR (826 nt, 3 exons, 52.67% GC) fall within the range of the global lncRNA distribution (length: 200–2400 nt, median ~800 nt; exon number: 1–6, median 3; GC content: 25–65%, median ~45%). These features support the use of LINC00998 and ANCR as representative reporters for studying spliced lncRNA export.

The TREX complex functions as a central hub that coordinates co-transcriptional assembly of export-competent mRNPs, and its role in bulk mRNA export has been well established across eukaryotes [34, 35]. Our nuclear lncRNPs purification followed by mass spectrometry analysis revealed enrichment of TREX components UAP56 and ALYREF. Depletion of UAP56 or TAP caused strong nuclear retention of both lncRNAs and β-globin mRNA, whereas depletion of ALYREF specifically inhibited the export of spliced lncRNAs with no apparent effect on the export of spliced β-globin mRNA, suggesting the export of spliced lncRNAs may not be exactly the same as spliced mRNAs.

Intriguingly, the EJC component eIF4A3 emerged as a critical factor for the export of two spliced lncRNAs tested. The EJC is deposited 20–24 nucleotides upstream of exon-exon junctions during splicing and serves as a binding platform for multiple factors involved in post-transcriptional RNA regulation [36–39]. Core components of the EJC include eIF4A3, Y14 (RBM8A), MAGOH/MAGOHB, and CASC3 (MLN51). The major function of EJC turns out to be in nonsense-mediated decay, whereas the early conclusion of EJC function in mRNA export is mostly based on the misclassification of UAP56 and ALYREF as major components of EJC [36, 40]. In this study, we showed that depletion of eIF4A3, Y14, or MAGOH/MAGOHB only resulted in significant nuclear retention of spliced lncRNAs with no apparent interference with the export of spliced β-globin mRNA, which further supports unique features for the export of spliced lncRNAs.

In contrast, CASC3, an EJC component enriched in the cytoplasm, was dispensable for the export of both lncRNAs and mRNAs. This observation was consistent with recent findings that the EJC underwent a compositional switch during its lifecycle, with CASC3 being recruited to the complex primarily in the cytoplasm after nuclear export was completed [41, 42]. The stable trimeric core comprising eIF4A3-Y14-MAGOH is formed when the Y14-MAGOH heterodimer inhibits the ATPase activity of eIF4A3 [43–45], thereby trapping the ATP-bound EJC onto spliced RNA in a stable conformation required for nuclear export.

The NPC components emerged as critical and differential regulators of lncRNA export. Our findings revealed a complex hierarchy of nucleoporin requirements that distinguishes lncRNA from mRNA export. NUP98 depletion caused strong nuclear retention of LINC00998 and ANCR with no effect on spliced β-globin and GADD45 mRNAs, suggesting NUP98 as a lncRNA-biased export factor. NUP98 is a mobile nucleoporin with transcription-dependent dynamics and localizes to both nuclear and cytoplasmic sides of the NPC [46, 47]. It contains FG/GLFG repeats that interact with multiple export factors, including TAP and RAE1, facilitating selective mRNP transit and forming an essential component of the nuclear pore permeability barrier [48–50]. Recent structural studies have revealed that the FG-repeat domain of NUP98 contains molecular interactions crucial for the phase separation properties of the nuclear pore permeability barrier [50]. The preference of NUP98 for lncRNA export likely reflects distinct biophysical properties of lncRNPs, such as size, charge distribution, or surface-exposed motifs that require more extensive NUP98 interactions compared to canonical mRNPs.

Similar to NUP98, depletion of NUP62, NUP93, NUP133, NUP153, NUP88, NUP214, RanBP2, NUP107, or ELYS also preferentially impaired lncRNA export without affecting spliced β-globin mRNA. In contrast, depletion of NUP160 and NUP205 showed much broader effects, impairing export of both lncRNAs and mRNAs. NUP160 and NUP205 are both structural nucleoporins that form the scaffold of the NPC and are critical for its assembly and function [51, 52]. The differential nucleoporin requirements between lncRNAs and mRNAs suggested that nuclear pore complex function is more complicated than previously appreciated, with substrate-specific pathways coexisting within the same physical structure. This broad nucleoporin requirement distinguishes lncRNA export from the more streamlined mRNA export pathway [48, 49]. One possibility is that lncRNAs utilize more NPC interaction sites during their transit, requiring contributions from multiple nucleoporins. Alternatively, lncRNAs may be more sensitive to alterations in NPC permeability barrier properties, making them dependent on the proper assembly and function of various nucleoporin subcomplexes. One limitation of this study is that the differential export phenotypes observed upon depletion of distinct NPC components may be influenced by variations in knockdown efficiency. Achieving comparable knockdown levels across different NPC subunits in future experiments will be necessary to validate the selective roles of individual NPC components in lncRNA export.

Transcriptome-wide analysis confirmed the selective requirements observed with reporter systems. eIF4A3 and NUP98 depletion predominantly affected lncRNA localization as compared to mRNAs. In striking contrast, NUP160 depletion impacted both classes, consistent with its role as a general structural component. The findings demonstrated that lncRNA export represents a distinct regulatory layer in gene expression, with EJC components and specific nucleoporins serving as selective gatekeepers. This selectivity may enable cells to independently regulate lncRNA and mRNA abundance in the cytoplasm, providing a mechanism to fine-tune gene regulatory networks without globally disrupting protein-coding gene expression.

Importantly, the lncRNA-biased nuclear retention observed under eIF4A3 and NUP98 knockdown showed no meaningful correlation with transcript architecture, including GC content, exon number, transcript length, or splice site strength, suggesting it reflects fundamental molecular differences between RNA classes rather than measurable sequence features. lncRNAs undergo less efficient splicing with fewer EJC deposits per transcript and largely lack cis-acting export elements, including m6A modifications and ALYREF/NXF1-recruiting sequences, that enable co-transcriptional TREX recruitment and mRNA export independence from EJC/NUP98 [53–55]. As discussed above, the selectivity of NUP98 and eIF4A3 for lncRNA export likely reflects these class-intrinsic differences, where mRNAs can utilize redundant export adaptors to bypass strict EJC/NUP98 dependence, whereas lncRNAs cannot. The contrasting behavior of NUP160, whose depletion impairs export of both lncRNAs and mRNAs in a GC content-dependent manner, suggests it operates through a distinct class-nonspecific export mechanism. Future studies will be needed to further dissect the molecular basis of this lncRNA-biased export dependency.

Mechanistically, we proposed that splicing deposits the EJC on lncRNAs, which serves as a platform for ALYREF recruitment. Studies have shown that ALYREF contains a short, conserved motif that directs its interaction with both the cap-binding complex and the EJC component eIF4A3, and that export complexes are located between the 5’ cap and the first EJC on spliced transcripts [21, 56]. ALYREF then facilitates TAP binding, enabling formation of export-competent lncRNPs. The slower kinetics of lncRNA export likely reflect additional RNP remodeling steps required to navigate through the nuclear pore, in contrast to mRNAs that can utilize redundant adaptors to bypass strict EJC dependence [56–58].

**Fig. 6:**
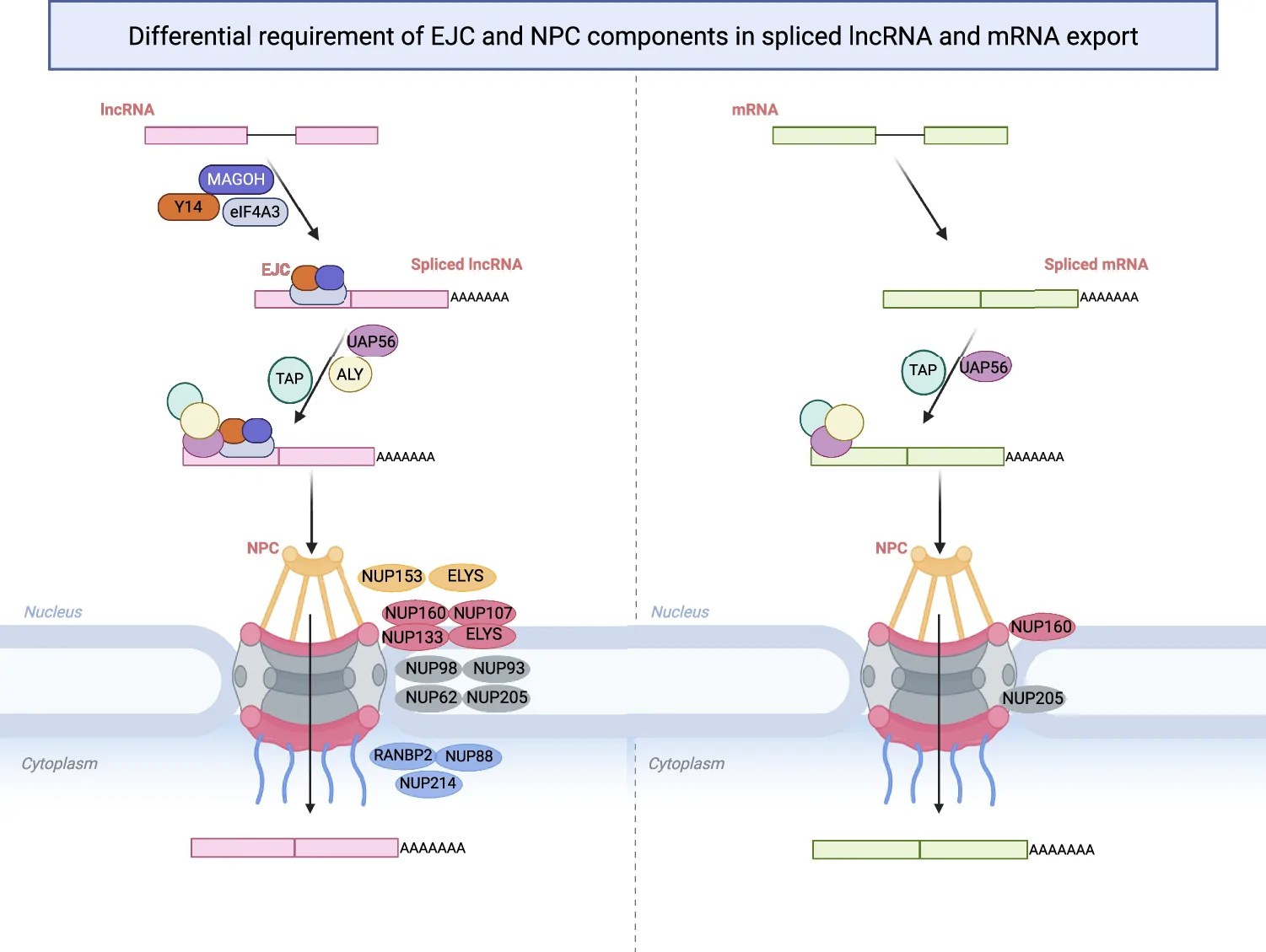
Model: EJC and NPC components preferentially regulate spliced lncRNA export. Spliced lncRNAs acquire EJC components (eIF4A3, Y14, MAGOH), recruit UAP56, ALYREF, and TAP, and transit through the NPC. A defined subset of nucleoporins, including NUP98, NUP160, NUP205, NUP62, NUP153, ELYS, NUP93, NUP88, NUP133, NUP107, NUP214, and RanBP2, preferentially support their export.

Collectively, our reporter-based and transcriptome-wide analyses support a hierarchical export model for spliced lncRNAs (Fig. 6). In this model, transit through the nuclear pore occurs via a specialized pathway that depends sequentially on the EJC trimeric core for RNP assembly in the nucleoplasm, on TREX-ALYREF for recruiting export adaptor TAP, and on an extended nucleoporin network (notably NUP98) for selective passage through the NPC. This multi-layered selectivity distinguishes lncRNA export from canonical mRNA export and reveals new regulatory opportunities for controlling lncRNA function through modulation of their cytoplasmic availability.

## Material and methods

### Constructs and antibodies

LINC00998 and ANCR were amplified by PCR using HeLa genomic DNA as the template and then cloned into the pcDNA3.1 vector at the KpnI/BamHI and KpnI/ApaI sites, respectively. Wild-type (WT) β-globin containing its natural introns was described previously [59]. GADD45-pCDH vector was a gift from Yang Wang’s laboratory in Dalian Medical University. GADD45 was then re-cloned into the pcDNA3.1 at the NheI/NotI sites. For RNP purification in vivo, LINC00998 and ANCR were recloned into the pcDNA5/FRT/TO vector containing three repeated MS2 hairpin sequences downstream of the multiple cloning site.

Rabbit polyclonal antibody against TAP (Proteintech, 10328-1-AP, 1:1000), XAB2 (Proteintech, 10637-1-AP, 1:800), NUP98 (Proteintech, 12329-1-AP, 1:1000), RAE1 (Proteintech, 20491-1-AP, 1:1000), eIF4A3 (Proteintech, 17504-1-AP, 1:1000), CASC3 (Proteintech, 18047-1-AP, 1:500), NUP160 (Proteintech, 16084-1-AP, 1:1000), NUP205 (Proteintech, 24439-1-AP, 1:5000), NUP62 (Proteintech, 13916-1-AP, 1:1000), NUP93 (Proteintech, 13077-1-AP, 1:1000), NUP133 (Proteintech, 12405-1-AP, 1:2000), RanBP2 (Proteintech, 27606-1-AP, 1:2000), NUP214 (Abclonal, A8357, 1:2000), NUP88 (Proteintech, 55465-1-AP, 1:1000), NUP153 (Proteintech, 14189-1-AP, 1:500), NUP107 (Proteintech, 19217-1-AP, 1:3000), ELYS (Proteintech, 30923-1-AP, 1:10,000), GAPDH (Proteintech, 10494-1-AP, 1:10,000), Rabbit monoclonal antibody against Y14 (Abcam, ab181038, 1:1000), mouse monoclonal antibodies against MAGOH (Proteintech, 68293-1-Ig, 1:10,000), and tubulin (Proteintech, 66031-1-Ig, 1:20,000) were used in western blot. Antibodies for ALYREF, PRPF19, UAP56, and CBP80 were gifts from Reed Lab in Harvard Medical School.

### Cell culture and transfection

HeLa, FRT-HeLa (a kind gift from Reed Lab, Harvard Medical School), and 293 T cells (American Type Culture Collection, USA) were maintained in Dulbecco’s Modified Eagle Medium (Gibco) supplemented with 10% fetal bovine serum (Gibco). For transient transfections, cells were cultured in 12-well plates and grown to ~80% confluence before transfection with 1 µg plasmid DNA using GoldenTran-DR (Golden Transfer Technology, China). For transient knockdown using RNA interference, cells were plated in 12-well plates and transfected at ~30% confluence with siRNAs using Lipofectamine 3000 (Invitrogen). A non-targeting siRNA served as the negative control. All siRNAs were purchased from RiboBio or GenePharma (China), and their sequences were provided in Suppl Table 3.

### Inducible stable knockdown cell lines

To establish DOX-inducible stable knockdown cell lines, shRNA sequences targeting eIF4A3, NUP98, and NUP160 were cloned into the pLKO-Tet-On inducible vector (Novartis, Switzerland). These plasmids were first packed with lentivirus proteins in 293 T cell line, and then the viruses were collected after 48 hours, and used to infect HeLa cells, followed by puromycin selection as described before [60]. The shRNA core sequences were listed in the Suppl Table 4.

### Microinjection

Reporter plasmids (1 µg) were co-injected with injection marker (Life, D7173) into the nuclei of HeLa cells. In each experiment, ~50 cells were microinjected, followed by incubation at 37 °C. After 30 min of incubation, α-amanitin (50 µg/mL) (Sigma) was added to inhibit further transcription. Cells were fixed at 0, 3, and 5 h for RNA fluorescence in situ hybridization (RNA-FISH).

### RNA-FISH

RNA-FISH was performed as previously described [61]. Briefly, cells were fixed 24 hours after plasmid transfection with 4% paraformaldehyde, followed by permeabilization with 0.1% Triton X-100. After washing with 1× SSC containing 50% formamide, cells were incubated with the probe overnight at 37 °C. A 70-nt DNA oligonucleotide complementary to the vector sequence downstream of the multiple cloning sites was used as the FISH probe. It was pre-labeled with Alexa Fluor 546 NHS ester at the 5′ end and purified by HPLC. The FISH probe sequence used was 5’-AAGGCACGGGGGAGGGGCAAACAACAGATGGCTGGCAACT AGAAGGCACAGTCGAGGCTGATCAGCGGGT. Nuclei were counterstained with DAPI. Fluorescence signals were visualized using the DMI8 microscope (Leica).

### Mini nuclear extract preparation

Nuclear and cytoplasmic fractionation was performed as previously described [62]. Briefly, HeLa cells at ~90% confluency were detached with a cell scraper and incubated in a hypotonic buffer. Cell lysis was achieved using a dounce homogenizer, and nuclei were subsequently isolated with sequential low- and high-salt extractions. Nuclear extracts were transferred into Ultra-0.5 mL Centrifugal Filters (Amicon) for concentration by centrifuging at 12,000 rpm. The nuclear fraction was then dialyzed in buffer (20 mM HEPES, 100 mM KCl, 0.2 mM EDTA, 20% glycerol) using MINI dialysis units (Thermo).

### Purification of RNPs assembled in vivo

Affinity purification of LINC00998 and ANCR lncRNPs formed in vivo was performed as previously described [63]. Briefly, the LINC00998 and ANCR plasmids in pcDNA5/FRT/TO vector containing three repeated MS2 hairpins downstream of each lncRNA, were co-transfected with pOG44 plasmid into FRT-HeLa cells to establish inducible stable cell lines. Nuclear extracts were prepared after 300 ng/mL DOX induction for 24 h, and RNPs assembled in vivo were then purified using MS2-MBP-based affinity purification. Purified lncRNPs were separated on SDS-PAGE and visualized by silver staining. Mass spectrometric analysis was conducted at the core facility of Tsinghua University.

### RNA sequencing and data analysis

Inducible stable knockdown cell lines were seeded in 12-well plates, with two wells per condition: one untreated control and one treated with DOX to a final concentration of 1 µg/mL for 72 h. After induction, cytoplasmic and nuclear fractions were separated using an extraction kit (Beyotime, China) according to the manufacturer’s instructions. RNA from each fraction was purified using TRIzol (Sigma), and separation efficiency was verified by RT-PCR using MALAT1 and RPS14 as nuclear and cytoplasmic markers, respectively.

For eIF4A3 and NUP98 knockdown samples, ribosomal RNA was removed using an rRNA-depletion strategy. For NUP160 knockdown samples, polyadenylated RNA was enriched using an Oligo(dT)-based poly(A) selection method. The resulting RNA was used for strand-specific library construction, including fragmentation, adaptor ligation, and size selection of 370–420 bp fragments, followed by RNA sequencing on the Illumina platform with PE150 strategy by Novogene (China). Transcript abundance was calculated using FPKM. LncRNA expression was quantified at the transcript level, and mRNA expression was quantified at the gene level.

To calculate the fraction of spliced and cytoplasmic polyA+ lncRNAs/mRNAs whose nuclear export were sensitive to eIF4A3/NUP98/NUP160 depletion, we first established a reference set of cytoplasm-localized polyA+ RNAs using NUP160 sequencing data, defined as those with FPKM NC_CE_ > FPKM NC_NE_, where NE and CE represented nuclear and cytoplasmic fractions, respectively. For eIF4A3 and NUP98, to minimize library preparation bias, the same criterion of cytoplasmic localization (FPKM NC_CE_ > FPKM NC_NE_) was applied to their respective sequencing data, and the resulting RNAs were intersected with the NUP160-defined reference set. Next, using the GENCODE human gene annotation (release 44, gencode.v44.annotation.gtf), exon numbers were retrieved for annotation, and RNAs with more than one exon were considered spliced. Nuclear retention score (NRS) was calculated as:

$${NRS}=log2\left(\frac{{FPKM}\,{{KD}}_{{NE}}}{{FPKM}\,{{KD}}_{{NE}}+{FPKM}\,{{KD}}_{{CE}}}\right)-log2\left(\frac{{FPKM}\,{{NC}}_{{NE}}}{{FPKM}\,{{NC}}_{{NE}}+{FPKM}\,{{NC}}_{{CE}}}\right)$$

where KD represented knockdown samples. Finally, only those lncRNA transcripts and mRNA genes with an NRS > 1 were classified as nuclear retained.

To characterize the transcript features of lncRNAs/mRNAs whose nuclear export were sensitive to eIF4A3/NUP98/NUP160 depletion, polyA+ RNAs were first defined using NUP160 sequencing data as those with FPKM NC_NE_ + FPKM NC_CE_ > 0. For eIF4A3 and NUP98, to minimize library preparation bias, all detected RNAs from their respective sequencing datasets were intersected with the NUP160-defined polyA+ RNA set. The nuclear-to-total RNA ratio (NTR) was calculated as NTR = FPKM_NE/(FPKM_NE + FPKM_CE). GC content, exon number, and transcript length were annotated based on the GENCODE gene annotation file. Splice site strength was quantified as previously described [64]. Briefly, multi-exon transcripts were identified from GENCODE annotation, intron coordinates were inferred from exon structures, and the corresponding 5′ and 3′ splice site sequences were extracted with BEDTools getfasta [65]. Splice site scores were then calculated using MaxEntScan [66] with 9-nt windows for 5′ splice sites (−3 to +6) and 23-nt windows for 3′ splice sites (−20 to +3). Site-level scores were further summarized into transcript-level metrics. Spearman correlation analysis was performed between log2(KD_NTR/NC_NTR) and each of the following features: GC content, exon number, transcript length, and splicing site strength.

### RT-PCR

Total RNA was purified using TRIzol (Sigma) and treated with DNase I (Promega). 1 µg of DNA-free total RNA was reverse transcribed using M-MLV reverse transcriptase (Promega). The PCR products were run on an agarose gel and the band intensity was quantified with ImageJ software. The primer sequences were listed in Suppl Table 5.

### Statistical analysis

Data from three independent experiments were presented as mean ± standard deviation. For RNA FISH quantification of the percentage of cells exhibiting cytoplasmic signal, comparisons between two groups were performed using an unpaired two-tailed Student’s *t* test, while experiments involving more than two groups were analyzed by one-way ANOVA. For analysis of endogenous RNA expression, multiple paired t-tests were performed with false discovery rate (FDR) correction using the two-stage step-up method of Benjamini, Krieger, and Yekutieli (*Q* = 1%). Statistical significance is indicated as *: *p* < 0.05, **: *p* < 0.01, and ***: *p* < 0.001. All statistical analyses and graph generation were performed using GraphPad Prism 10.

## Supplementary information

Supplementary Figure legends

Supplementary Figure 1

Supplementary Figure 2

Supplementary Figure 3

Supplementary Figure 4

Supplementary Figure 5

Supplementary Figure 6

Supplementary Figure 7

Supplementary Figure 8

Supplementary Figure 9

Supplementary Figure 10

Supplementary Figure 11

Supplementary Figure 12

Supplementary Figure 13

Supplementary Table 1

Supplementary Table 2

Supplementary Table 3

Supplementary Table 4

Supplementary Table 5

Original WB

## Data Availability

The data that support this study are available from the corresponding author upon request.
