# Supplementary material for: Exon junction complex and nuclear pore complex components preferentially govern the nuclear export of spliced lncRNAs
Source: Cell Death Discov. 2026 Jul 8;12:392. doi: 10.1038/s41420-026-03246-0 (PMC13631367; doi:10.1038/s41420-026-03246-0)
Supplement: Supplementary file 1 [file 41420_2026_3246_MOESM10_ESM.pdf]

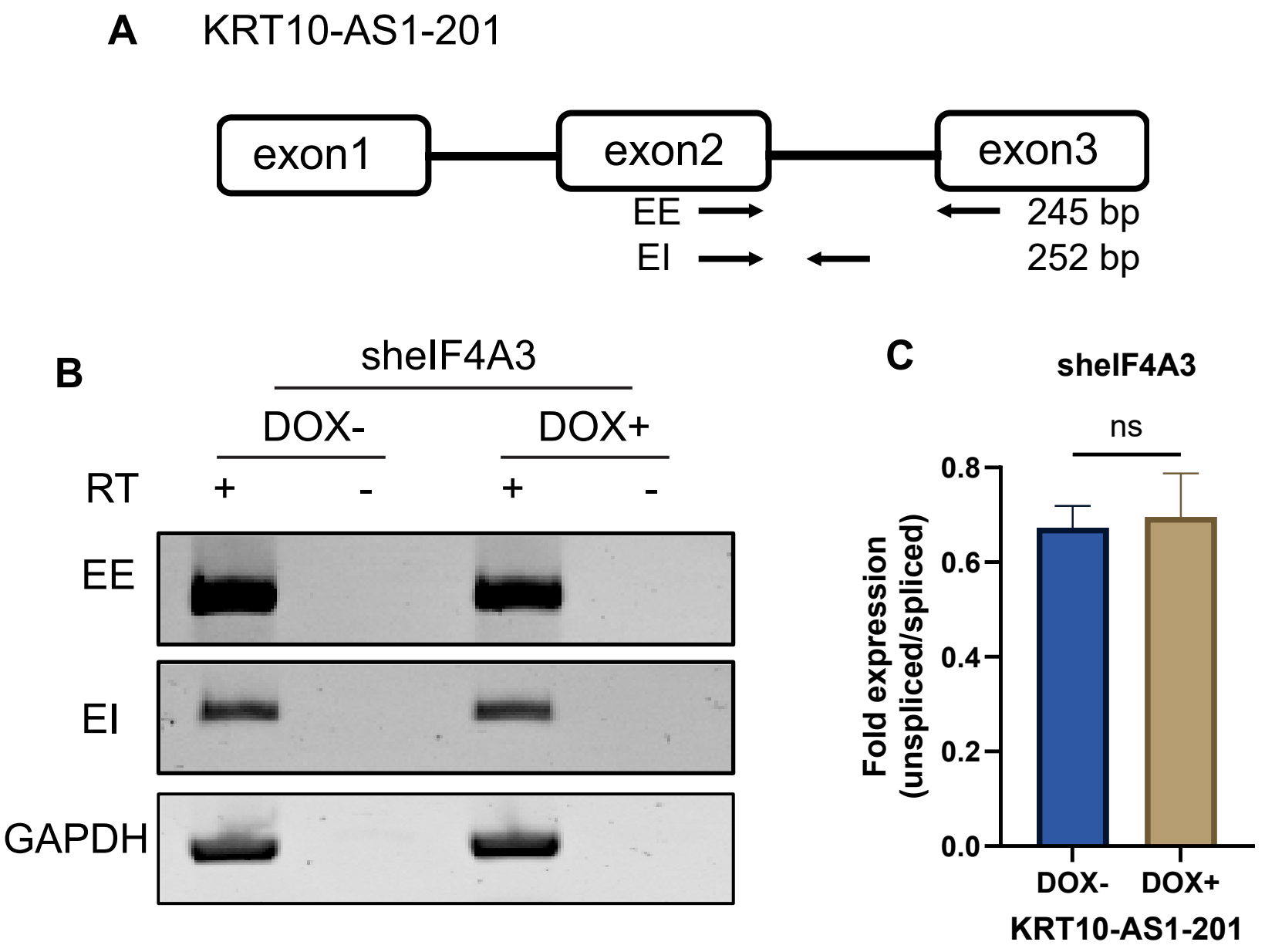

A
KRT10-AS1-201
exon1
exon2
exon3
EE
245 bp
EI
252 bp
B
sheIF4A3
DOX-
DOX+
RT
+
-
+
-
EE
EI
GAPDH
C
sheIF4A3
ns
Fold expression (unspliced/spliced)
0.0
0.2
0.4
0.6
0.8
DOX-
DOX+
KRT10-AS1-201
