# Supplementary material for: Exon junction complex and nuclear pore complex components preferentially govern the nuclear export of spliced lncRNAs
Source: Cell Death Discov. 2026 Jul 8;12:392. doi: 10.1038/s41420-026-03246-0 (PMC13631367; doi:10.1038/s41420-026-03246-0)
Supplement: Supplementary file 2 [file 41420_2026_3246_MOESM11_ESM.pdf]

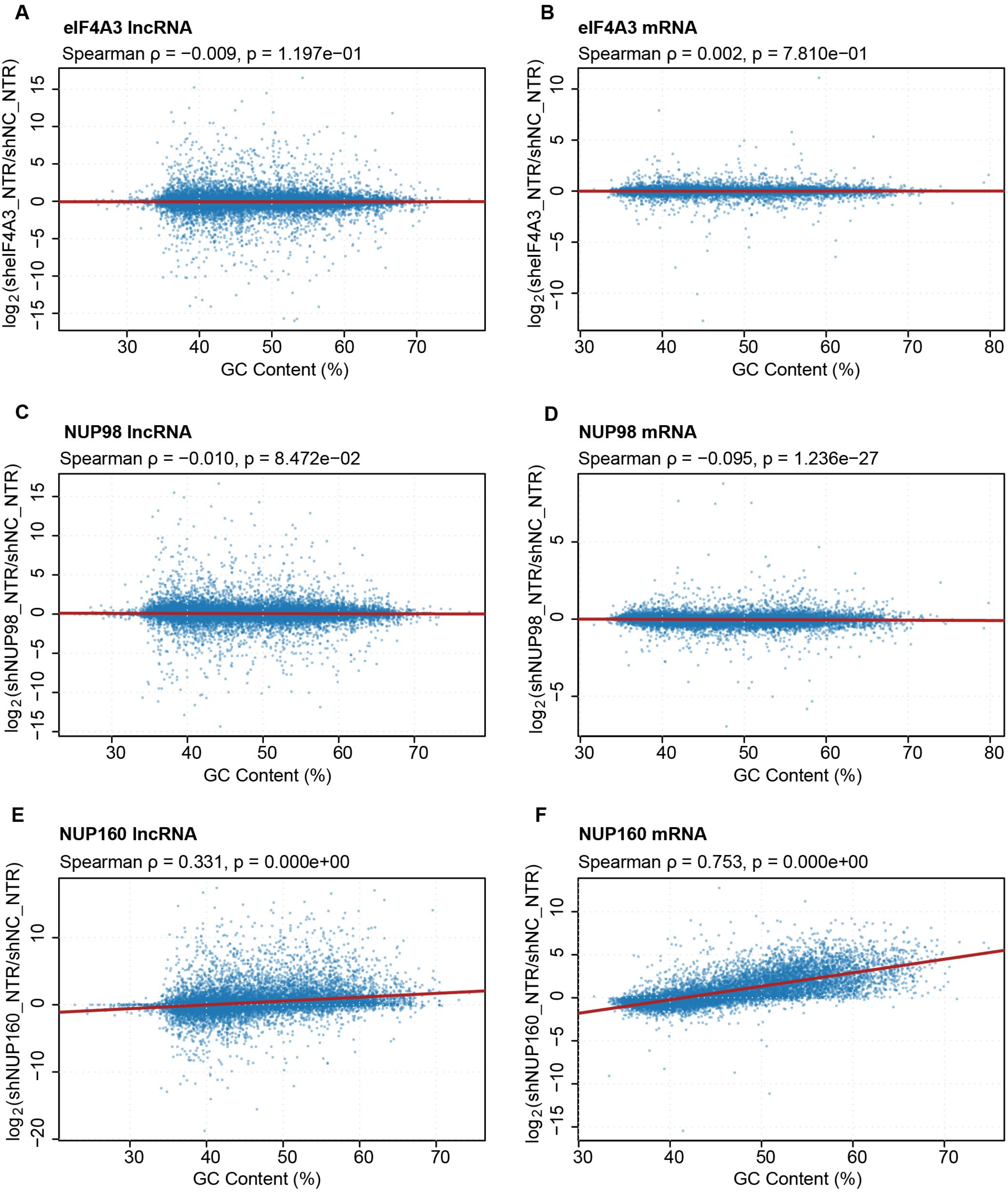
A
eIF4A3 lncRNA
Spearman ρ = −0.009, p = 1.197e−01
log2(sheIF4A3_NTR/shNC_NTR)
GC Content (%)
B
eIF4A3 mRNA
Spearman ρ = 0.002, p = 7.810e−01
log2(sheIF4A3_NTR/shNC_NTR)
GC Content (%)
C
NUP98 lncRNA
Spearman ρ = −0.010, p = 8.472e−02
log2(shNUP98_NTR/shNC_NTR)
GC Content (%)
D
NUP98 mRNA
Spearman ρ = −0.095, p = 1.236e−27
log2(shNUP98_NTR/shNC_NTR)
GC Content (%)
E
NUP160 lncRNA
Spearman ρ = 0.331, p = 0.000e+00
log2(shNUP160_NTR/shNC_NTR)
GC Content (%)
F
NUP160 mRNA
Spearman ρ = 0.753, p = 0.000e+00
log2(shNUP160_NTR/shNC_NTR)
GC Content (%)
