# Supplementary material for: Exon junction complex and nuclear pore complex components preferentially govern the nuclear export of spliced lncRNAs
Source: Cell Death Discov. 2026 Jul 8;12:392. doi: 10.1038/s41420-026-03246-0 (PMC13631367; doi:10.1038/s41420-026-03246-0)
Supplement: Supplementary file 3 [file 41420_2026_3246_MOESM12_ESM.pdf]

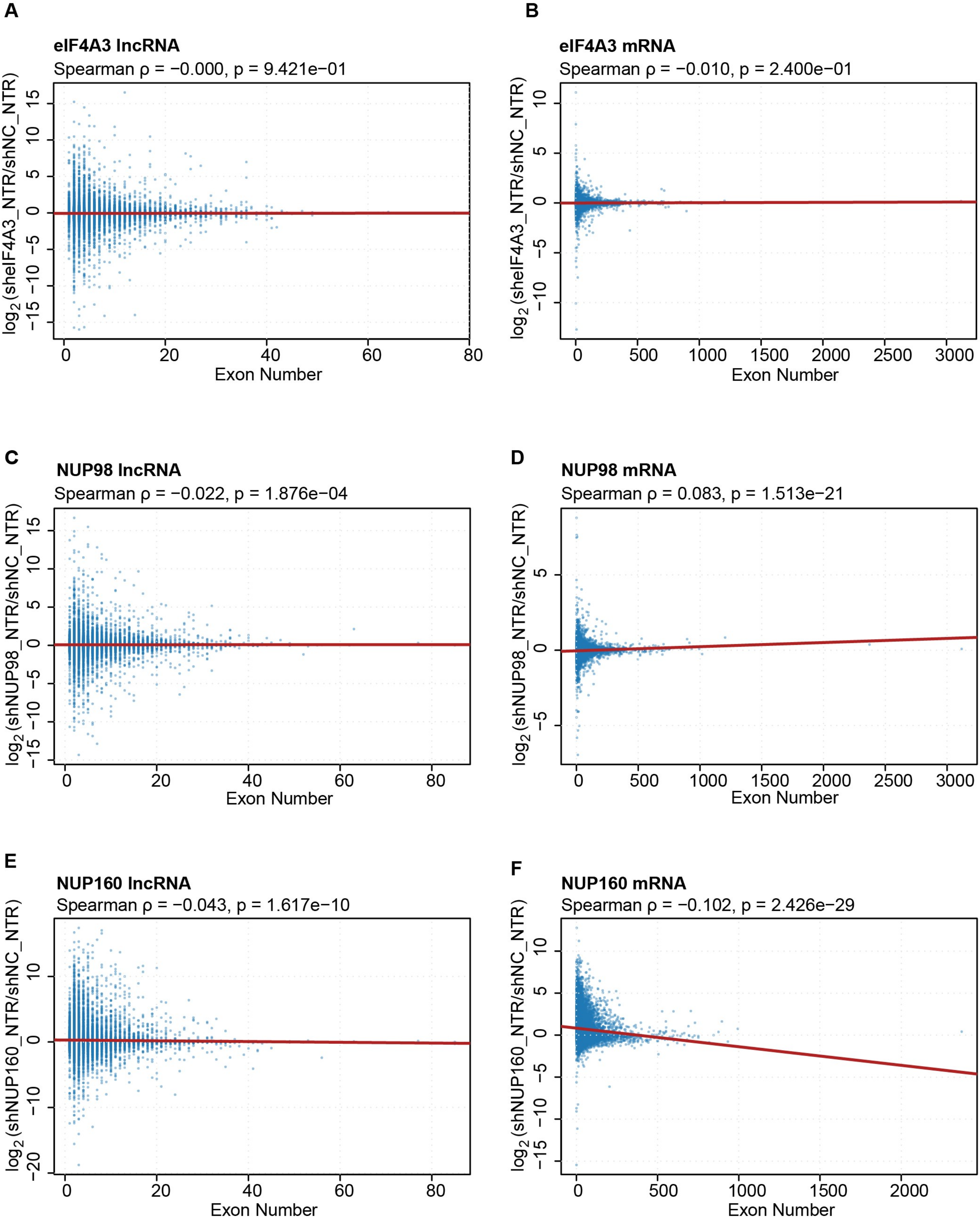
A
eIF4A3 lncRNA
Spearman ρ = −0.000, p = 9.421e−01
log2(sheIF4A3_NTR/shNC_NTR)
Exon Number
B
eIF4A3 mRNA
Spearman ρ = −0.010, p = 2.400e−01
log2(sheIF4A3_NTR/shNC_NTR)
Exon Number
C
NUP98 lncRNA
Spearman ρ = −0.022, p = 1.876e−04
log2(shNUP98_NTR/shNC_NTR)
Exon Number
D
NUP98 mRNA
Spearman ρ = 0.083, p = 1.513e−21
log2(shNUP98_NTR/shNC_NTR)
Exon Number
E
NUP160 lncRNA
Spearman ρ = −0.043, p = 1.617e−10
log2(shNUP160_NTR/shNC_NTR)
Exon Number
F
NUP160 mRNA
Spearman ρ = −0.102, p = 2.426e−29
log2(shNUP160_NTR/shNC_NTR)
Exon Number
