# Supplementary material for: Exon junction complex and nuclear pore complex components preferentially govern the nuclear export of spliced lncRNAs
Source: Cell Death Discov. 2026 Jul 8;12:392. doi: 10.1038/s41420-026-03246-0 (PMC13631367; doi:10.1038/s41420-026-03246-0)
Supplement: Supplementary file 4 [file 41420_2026_3246_MOESM13_ESM.pdf]

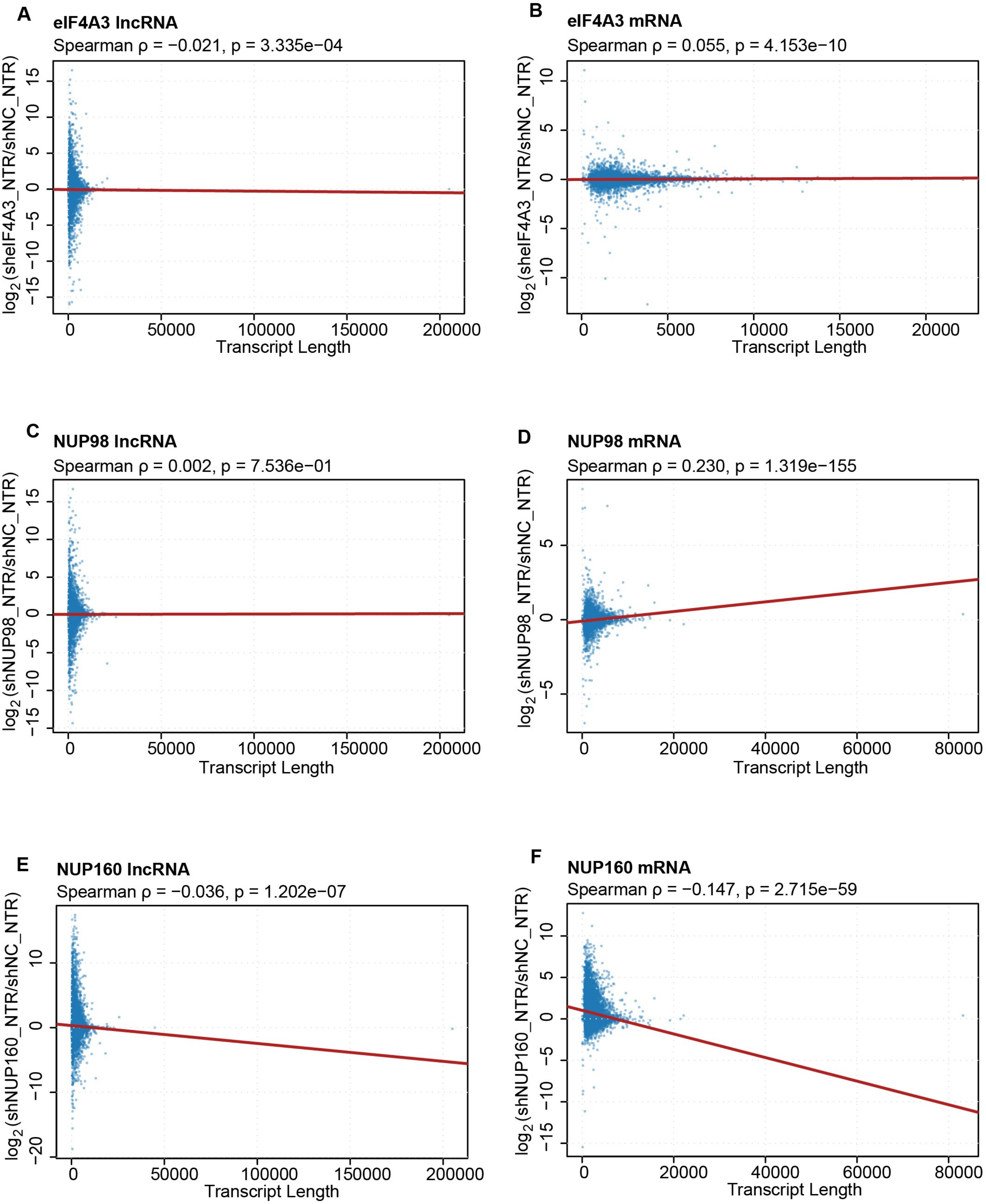
A
eIF4A3 lncRNA
Spearman ρ = −0.021, p = 3.335e−04
log2(sheIF4A3_NTR/shNC_NTR)
Transcript Length
B
eIF4A3 mRNA
Spearman ρ = 0.055, p = 4.153e−10
log2(sheIF4A3_NTR/shNC_NTR)
Transcript Length
C
NUP98 lncRNA
Spearman ρ = 0.002, p = 7.536e−01
log2(shNUP98_NTR/shNC_NTR)
Transcript Length
D
NUP98 mRNA
Spearman ρ = 0.230, p = 1.319e−155
log2(shNUP98_NTR/shNC_NTR)
Transcript Length
E
NUP160 lncRNA
Spearman ρ = −0.036, p = 1.202e−07
log2(shNUP160_NTR/shNC_NTR)
Transcript Length
F
NUP160 mRNA
Spearman ρ = −0.147, p = 2.715e−59
log2(shNUP160_NTR/shNC_NTR)
Transcript Length
