# Supplementary material for: Exon junction complex and nuclear pore complex components preferentially govern the nuclear export of spliced lncRNAs
Source: Cell Death Discov. 2026 Jul 8;12:392. doi: 10.1038/s41420-026-03246-0 (PMC13631367; doi:10.1038/s41420-026-03246-0)
Supplement: Supplementary file 5 [file 41420_2026_3246_MOESM14_ESM.pdf]

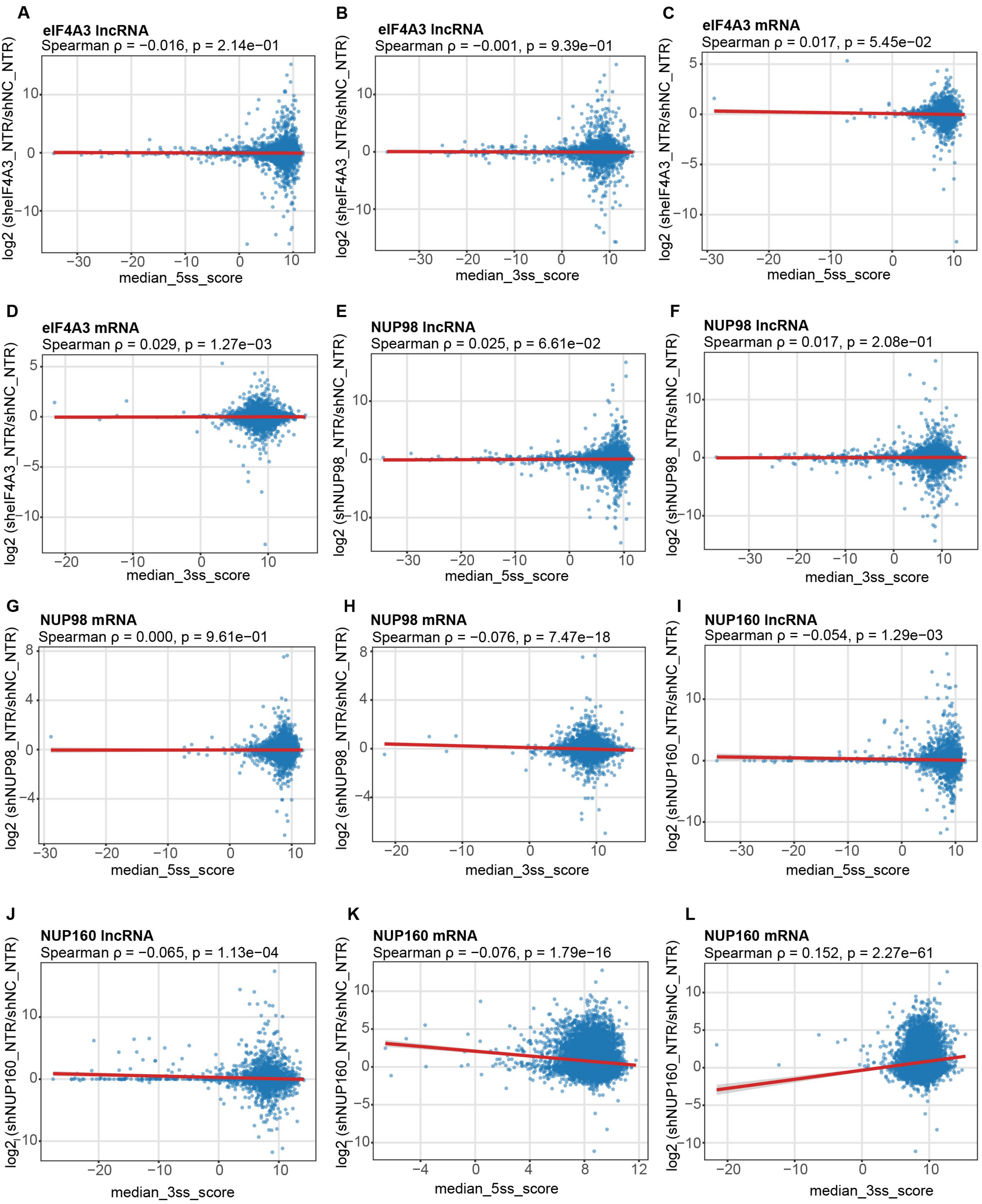
A
eIF4A3 lncRNA
Spearman ρ = −0.016, p = 2.14e−01
log2 (sheIF4A3_NTR/shNC_NTR)
median_5ss_score
B
eIF4A3 lncRNA
Spearman ρ = −0.001, p = 9.39e−01
log2 (sheIF4A3_NTR/shNC_NTR)
median_3ss_score
C
eIF4A3 mRNA
Spearman ρ = 0.017, p = 5.45e−02
log2 (sheIF4A3_NTR/shNC_NTR)
median_5ss_score
D
eIF4A3 mRNA
Spearman ρ = 0.029, p = 1.27e−03
log2 (sheIF4A3_NTR/shNC_NTR)
median_3ss_score
E
NUP98 lncRNA
Spearman ρ = 0.025, p = 6.61e−02
log2 (shNUP98_NTR/shNC_NTR)
median_5ss_score
F
NUP98 lncRNA
Spearman ρ = 0.017, p = 2.08e−01
log2 (shNUP98_NTR/shNC_NTR)
median_3ss_score
G
NUP98 mRNA
Spearman ρ = 0.000, p = 9.61e−01
log2 (shNUP98_NTR/shNC_NTR)
median_5ss_score
H
NUP98 mRNA
Spearman ρ = −0.076, p = 7.47e−18
log2 (shNUP98_NTR/shNC_NTR)
median_3ss_score
I
NUP160 lncRNA
Spearman ρ = −0.054, p = 1.29e−03
log2 (shNUP160_NTR/shNC_NTR)
median_5ss_score
J
NUP160 lncRNA
Spearman ρ = −0.065, p = 1.13e−04
log2 (shNUP160_NTR/shNC_NTR)
median_3ss_score
K
NUP160 mRNA
Spearman ρ = −0.076, p = 1.79e−16
log2 (shNUP160_NTR/shNC_NTR)
median_5ss_score
L
NUP160 mRNA
Spearman ρ = 0.152, p = 2.27e−61
log2 (shNUP160_NTR/shNC_NTR)
median_3ss_score
