# Supplementary material for: Exon junction complex and nuclear pore complex components preferentially govern the nuclear export of spliced lncRNAs
Source: Cell Death Discov. 2026 Jul 8;12:392. doi: 10.1038/s41420-026-03246-0 (PMC13631367; doi:10.1038/s41420-026-03246-0)

Figure 1

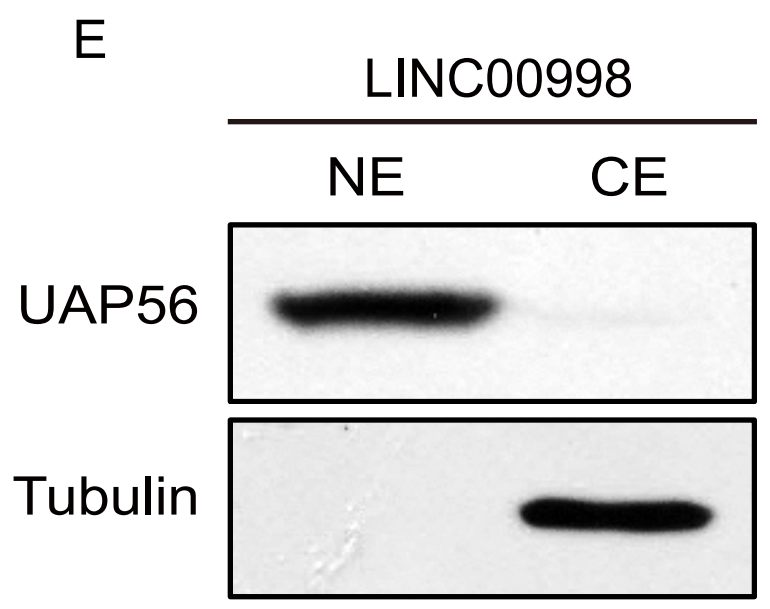
E
LINC00998
NE
CE
UAP56
Tubulin

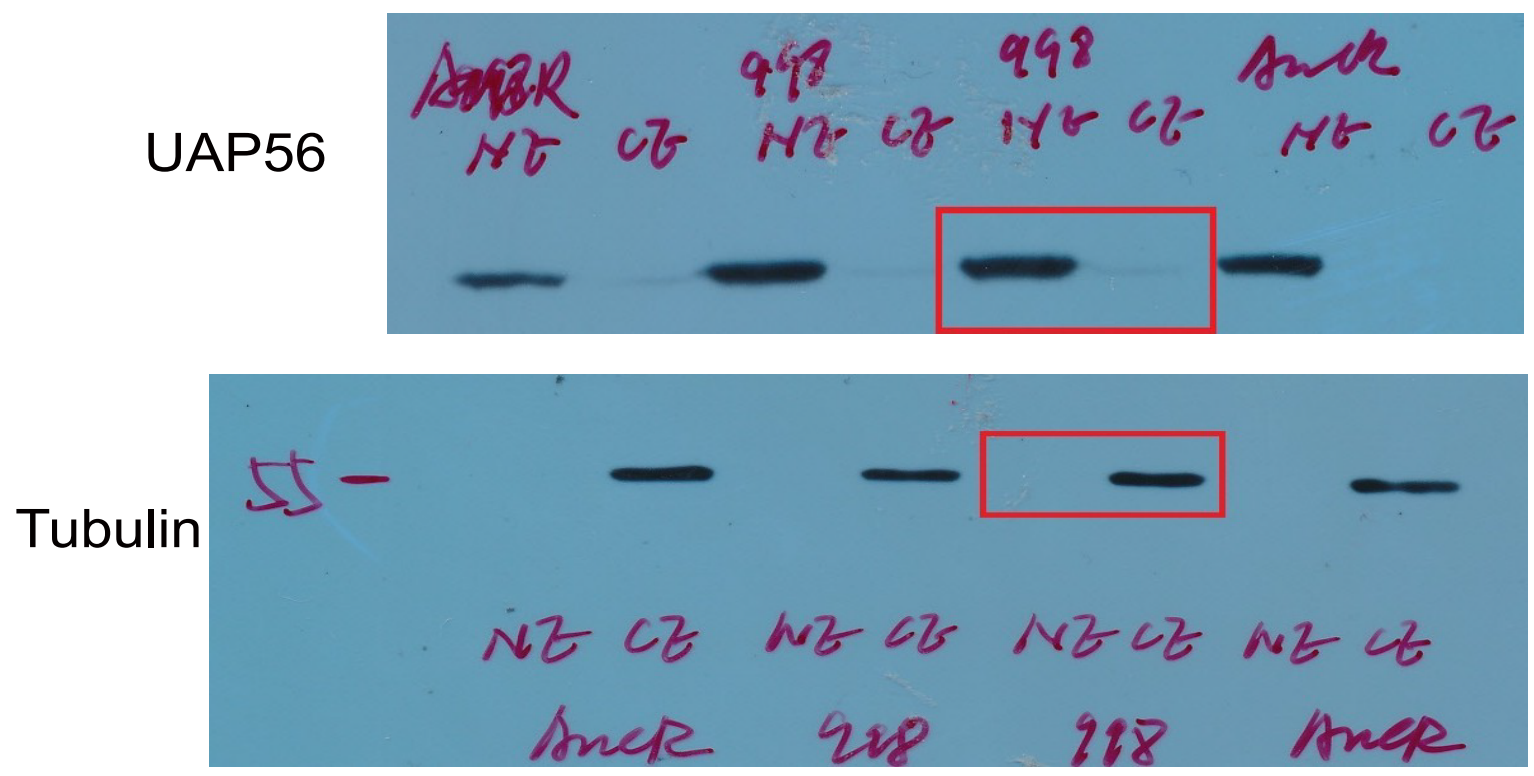
UAP56
Tubulin

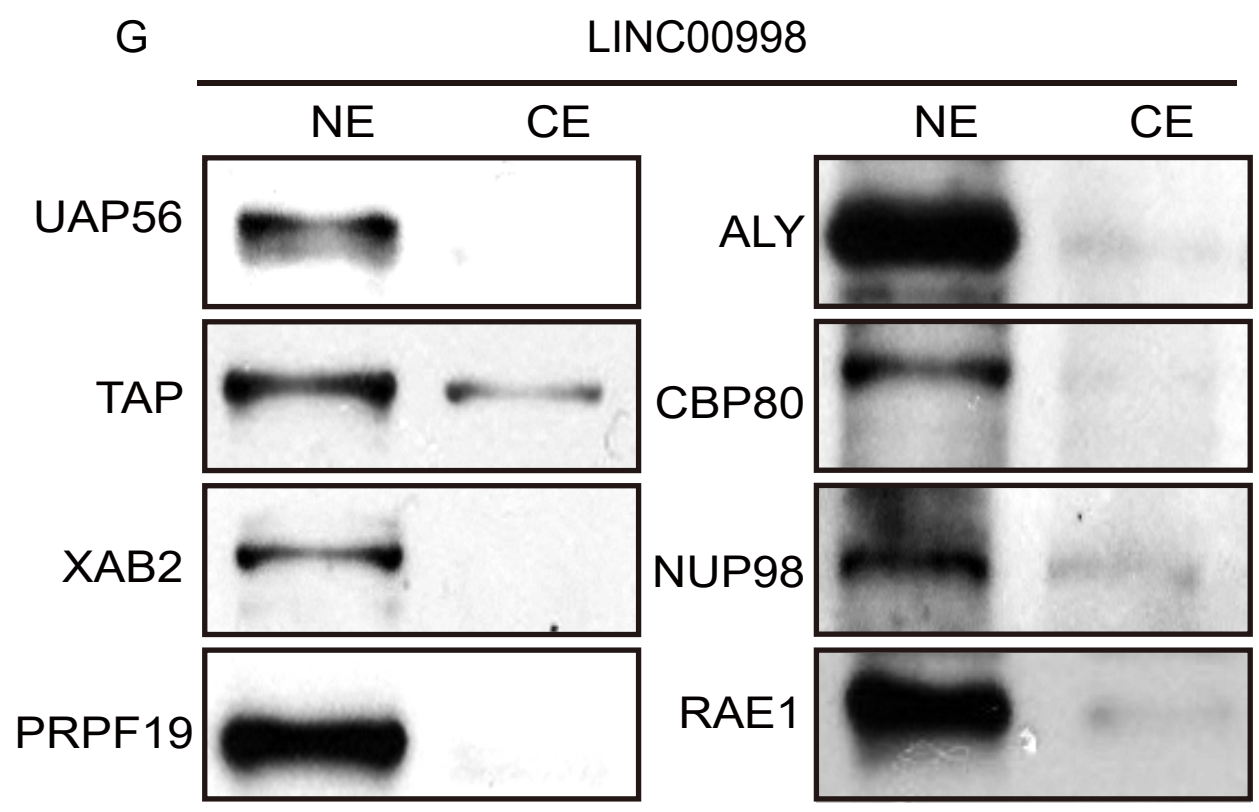
G
LINC00998
NE
CE
NE
CE
UAP56
TAP
XAB2
PRPF19
ALY
CBP80
NUP98
RAE1

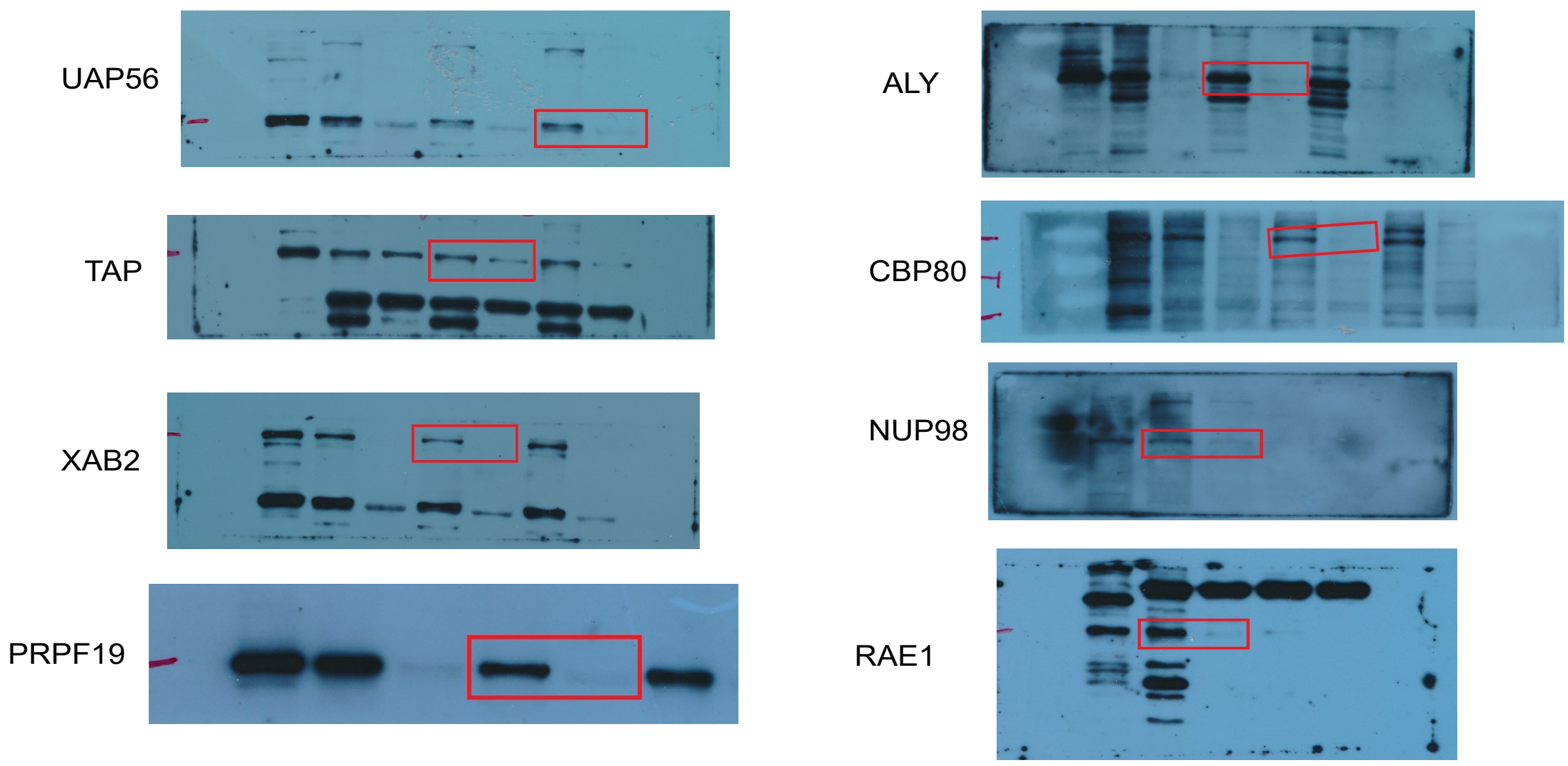
UAP56
TAP
XAB2
PRPF19
ALY
CBP80
NUP98
RAE1

Figure 2

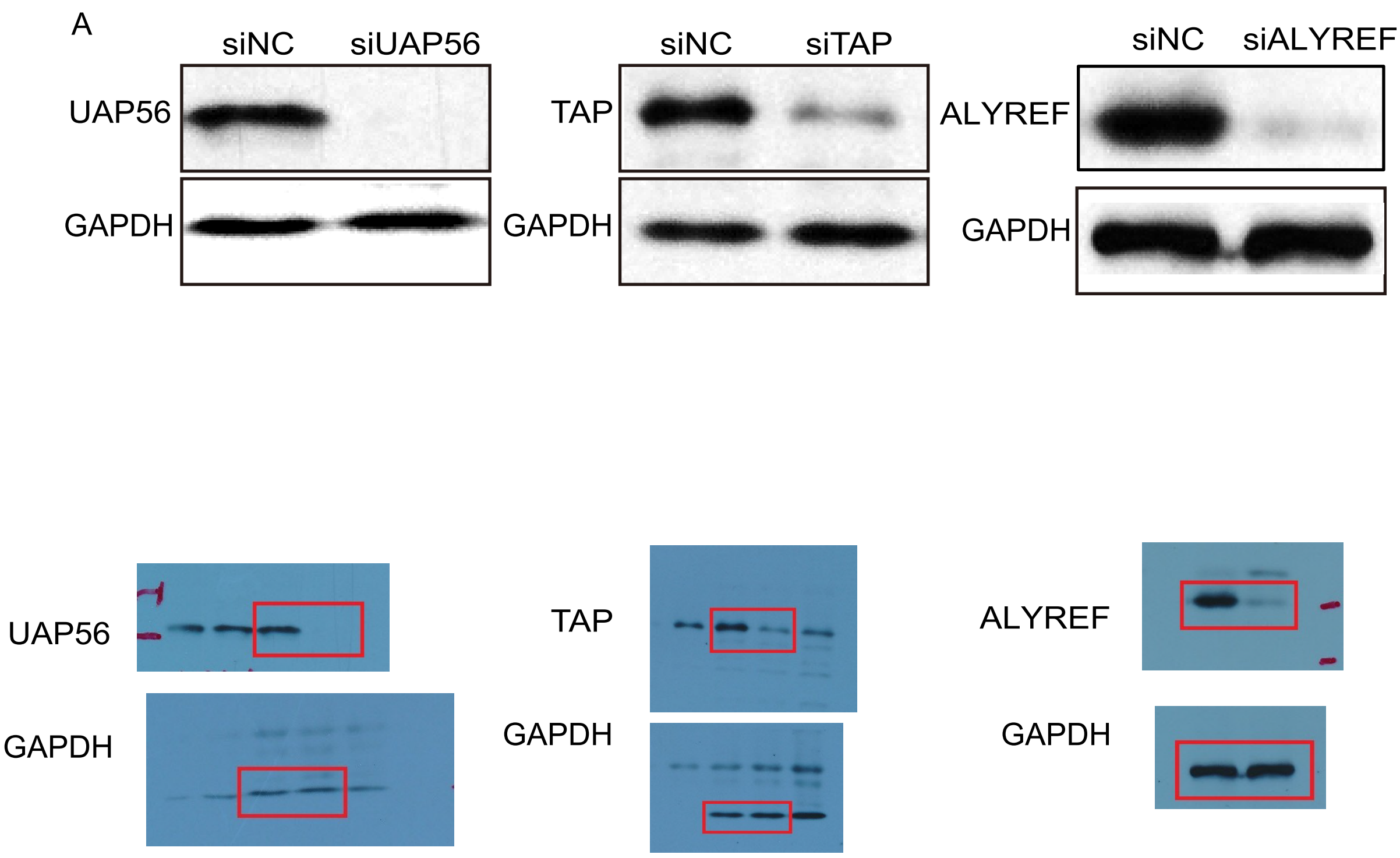
A
siNC
siUAP56
UAP56
GAPDH
siNC
siTAP
TAP
GAPDH
siNC
siALYREF
ALYREF
GAPDH
UAP56
GAPDH
TAP
GAPDH
ALYREF
GAPDH

Figure 3

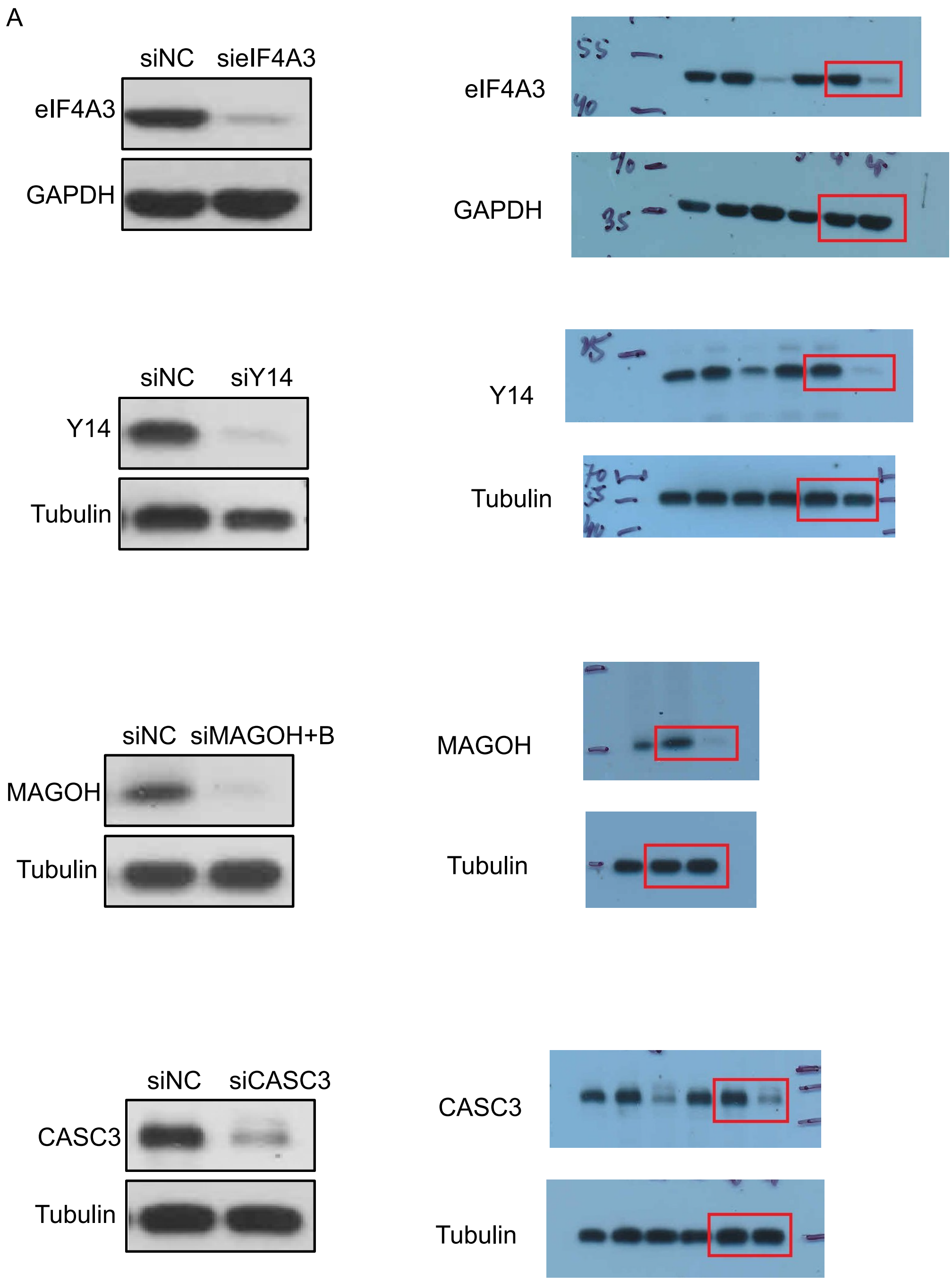

A
siNC
sieIF4A3
eIF4A3
GAPDH
siNC
siY14
Y14
Tubulin
siNC
siMAGOH+B
MAGOH
Tubulin
siNC
siCASC3
CASC3
Tubulin
eIF4A3
GAPDH
Y14
Tubulin
MAGOH
Tubulin
CASC3
Tubulin

Figure 4

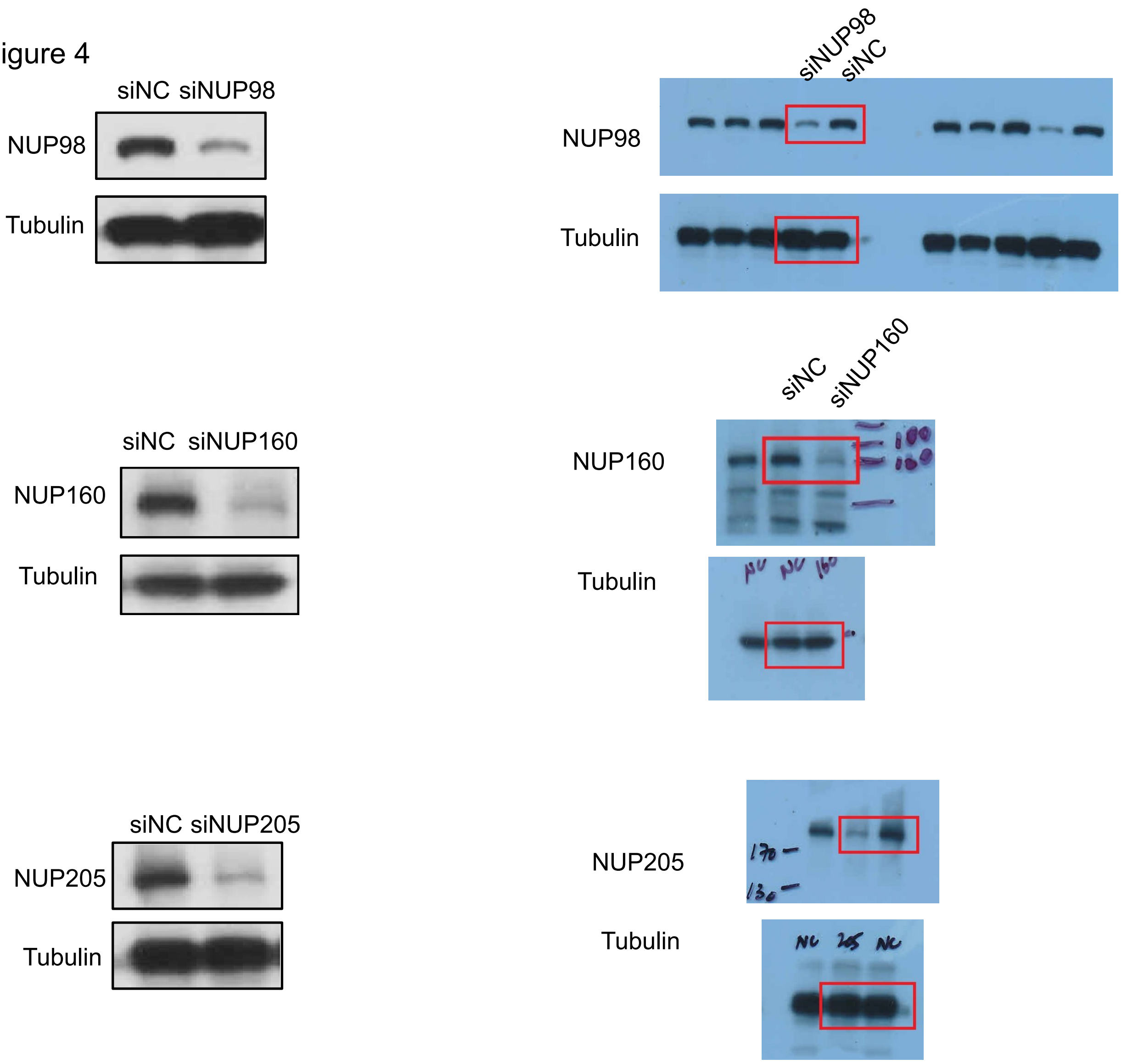
siNC siNUP98
NUP98
Tubulin
siNUP98
siNC
NUP98
Tubulin
siNC siNUP160
NUP160
Tubulin
siNC
siNUP160
NUP160
Tubulin
siNC siNUP205
NUP205
Tubulin
NUP205
Tubulin

Figure 5

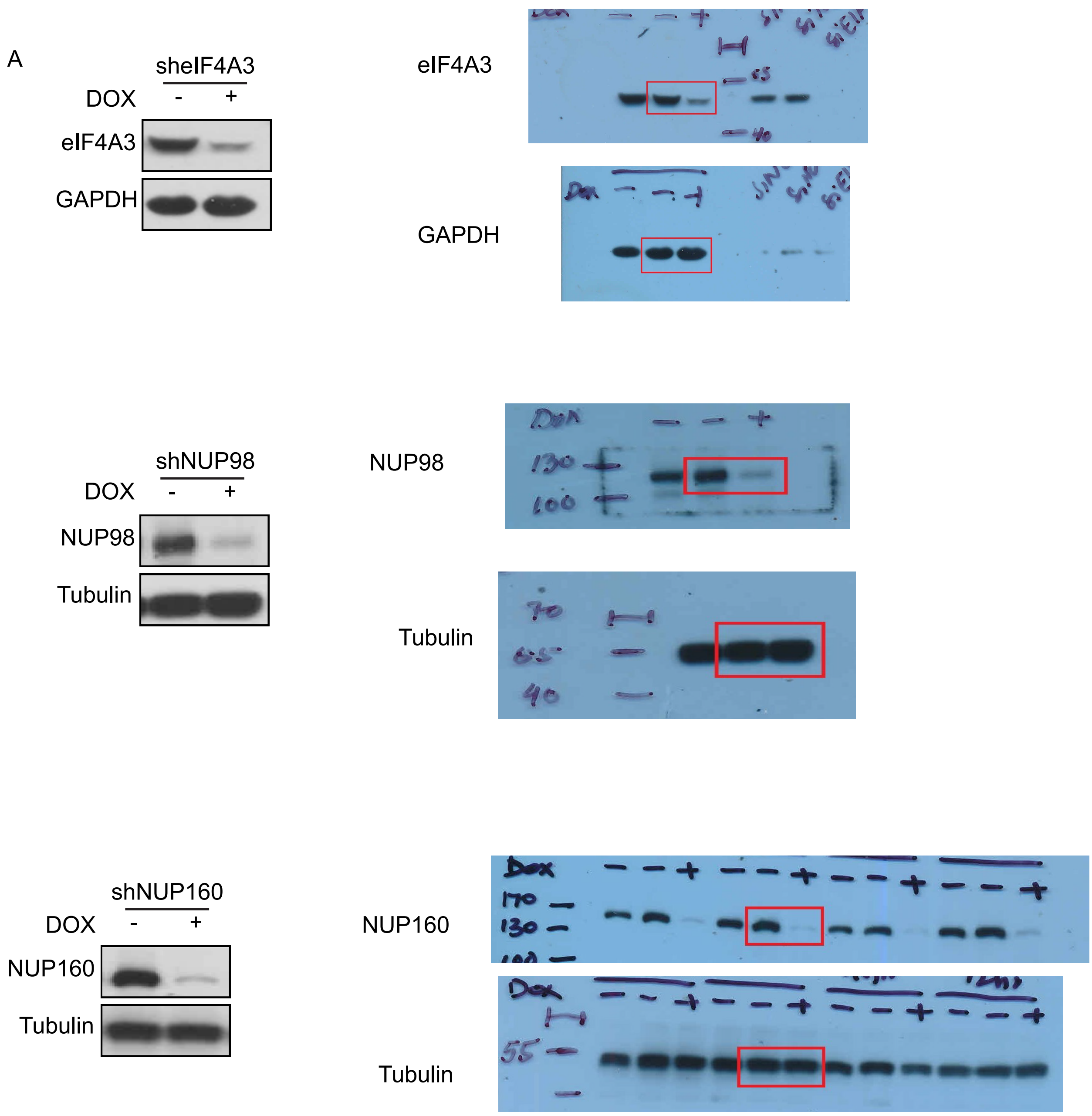
A
sheIF4A3
DOX - +
eIF4A3
GAPDH
eIF4A3
GAPDH
shNUP98
DOX - +
NUP98
Tubulin
NUP98
Tubulin
shNUP160
DOX - +
NUP160
Tubulin
NUP160
Tubulin

Supplementary Figure 1

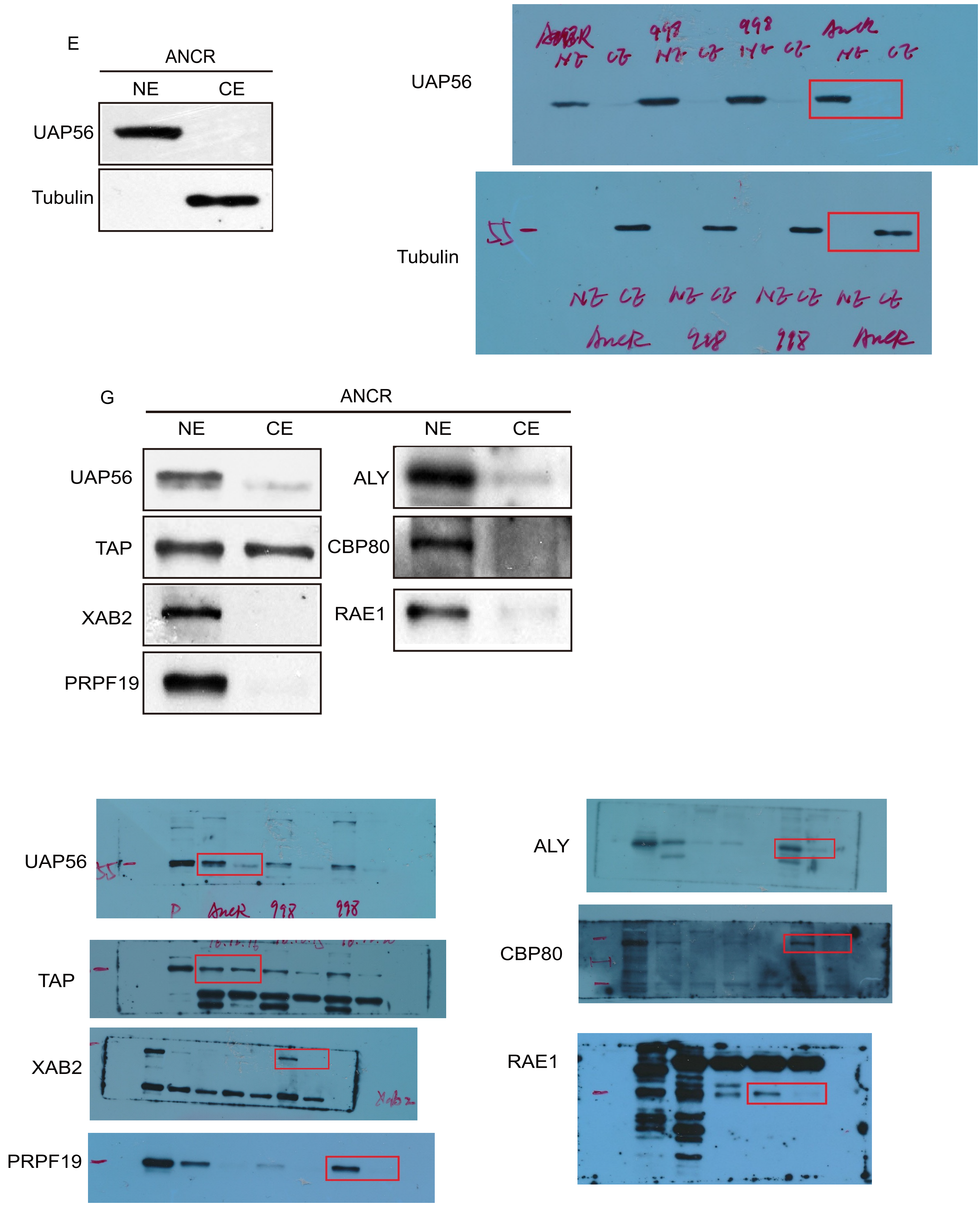

Supplementary Figure 4

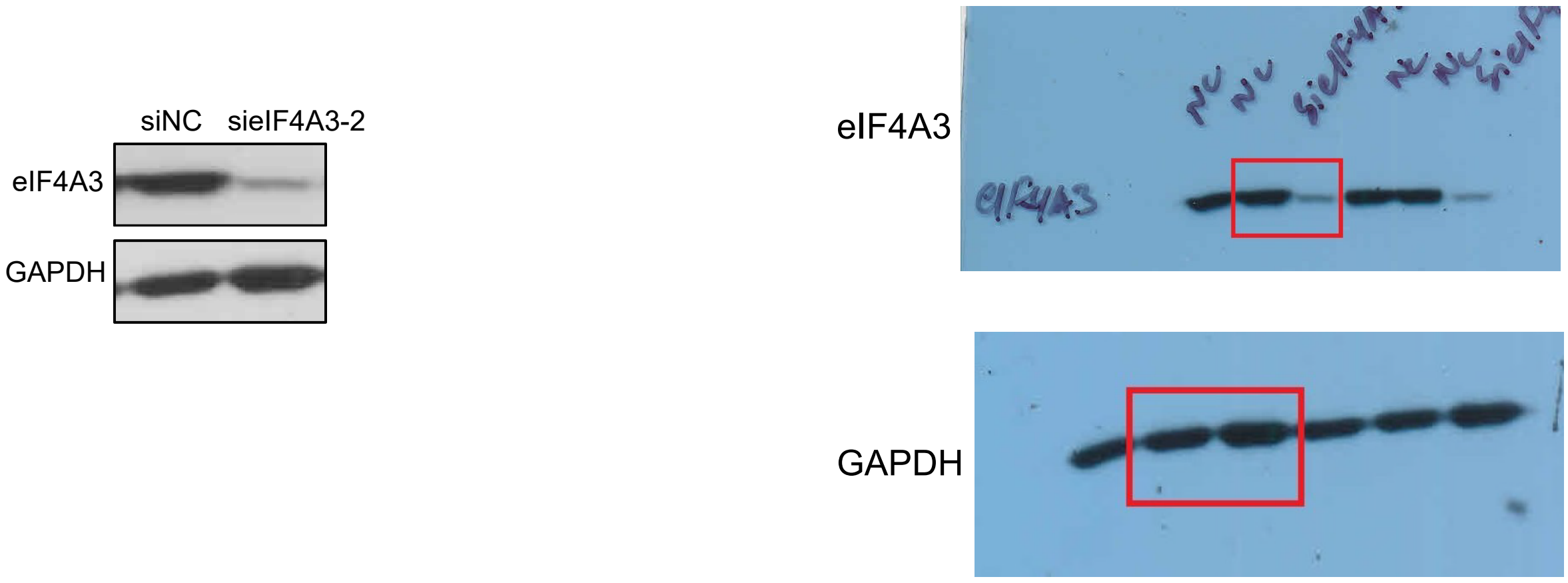

Supplementary Figure 6

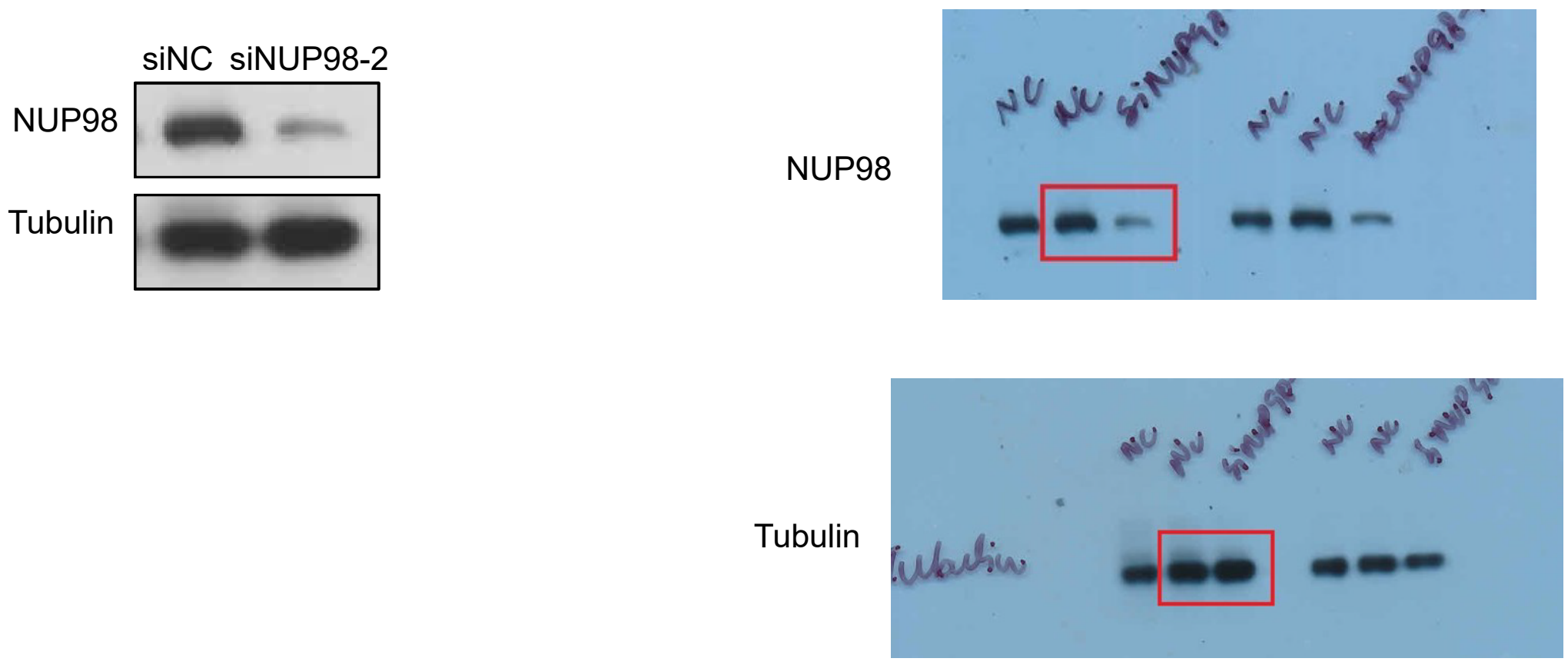

Supplementary Figure 7

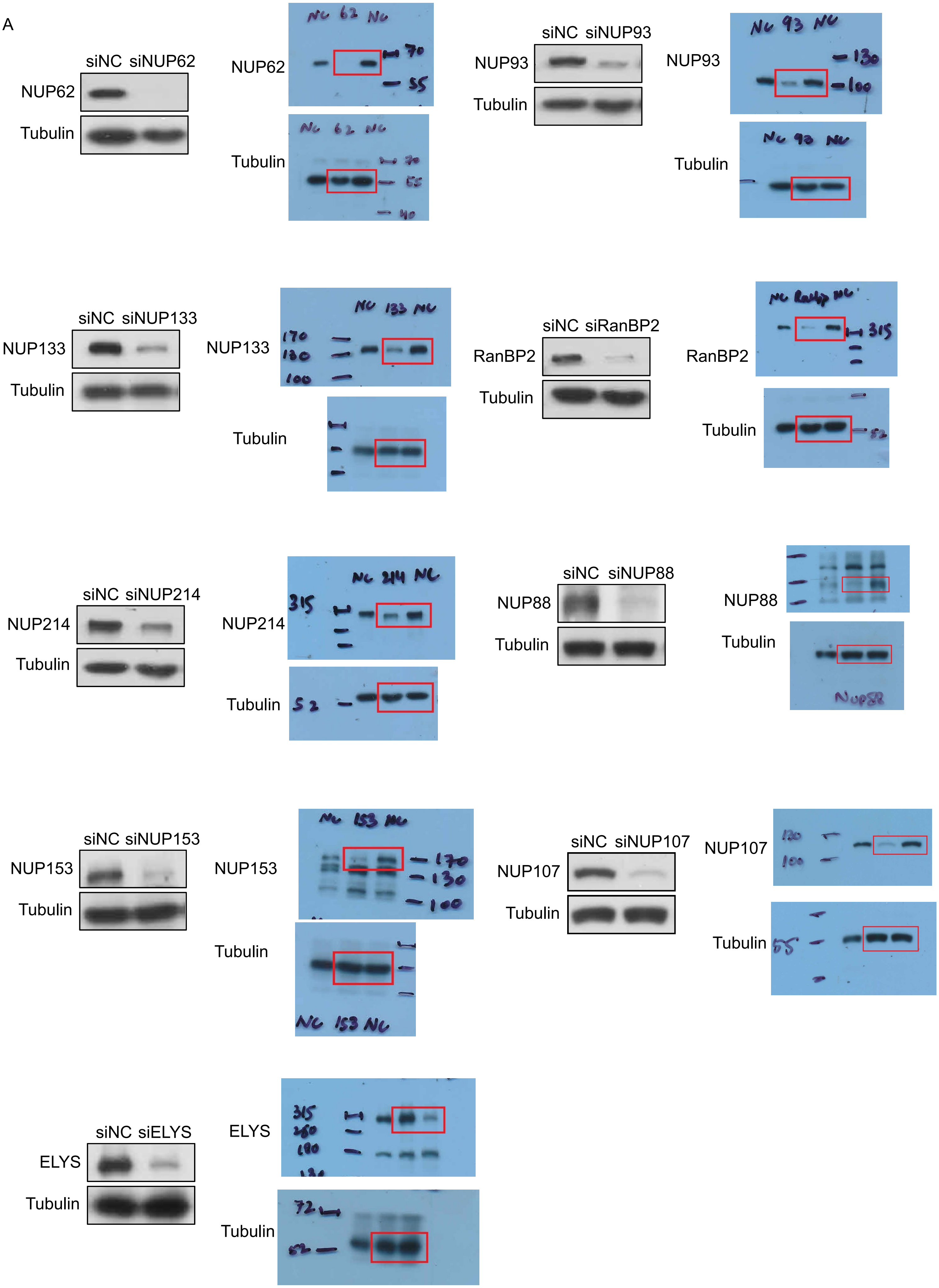

Supplement: Supplementary file 6 [file 41420_2026_3246_MOESM20_ESM.pdf]
