# Supplementary material for: Exon junction complex and nuclear pore complex components preferentially govern the nuclear export of spliced lncRNAs
Source: Cell Death Discov. 2026 Jul 8;12:392. doi: 10.1038/s41420-026-03246-0 (PMC13631367; doi:10.1038/s41420-026-03246-0)
Supplement: Supplementary file 7 [file 41420_2026_3246_MOESM2_ESM.pdf]

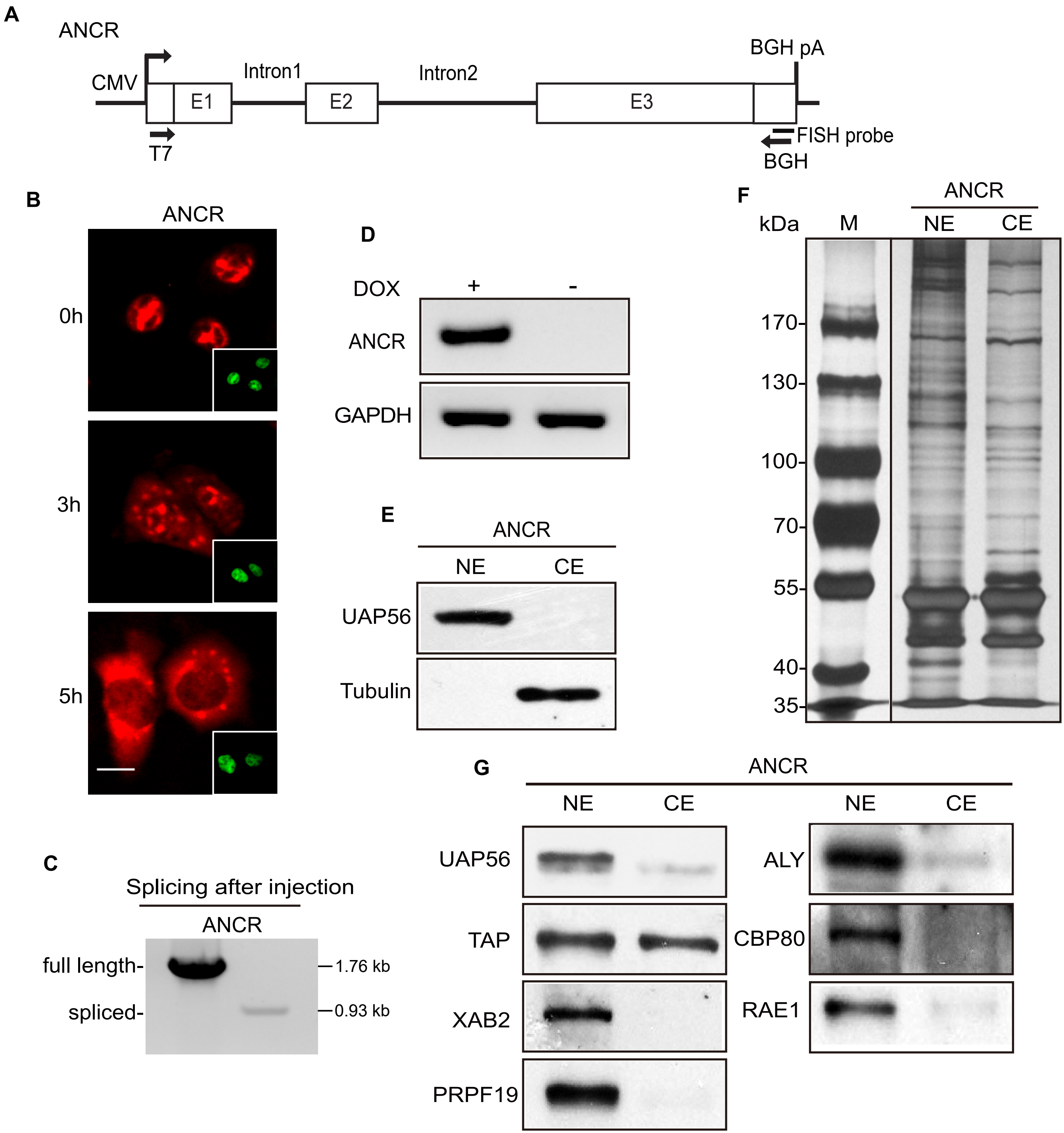
A
ANCR
CMV
E1
Intron1
E2
Intron2
E3
BGH pA
T7
FISH probe
BGH
B
ANCR
0h
3h
5h
C
Splicing after injection
ANCR
full length-
spliced-
1.76 kb
0.93 kb
D
DOX
+
-
ANCR
GAPDH
E
ANCR
NE
CE
UAP56
Tubulin
F
ANCR
kDa
M
NE
CE
170
130
100
70
55
40
35
G
ANCR
NE
CE
NE
CE
UAP56
TAP
XAB2
PRPF19
ALY
CBP80
RAE1
