# Supplementary material for: Exon junction complex and nuclear pore complex components preferentially govern the nuclear export of spliced lncRNAs
Source: Cell Death Discov. 2026 Jul 8;12:392. doi: 10.1038/s41420-026-03246-0 (PMC13631367; doi:10.1038/s41420-026-03246-0)
Supplement: Supplementary file 8 [file 41420_2026_3246_MOESM3_ESM.pdf]

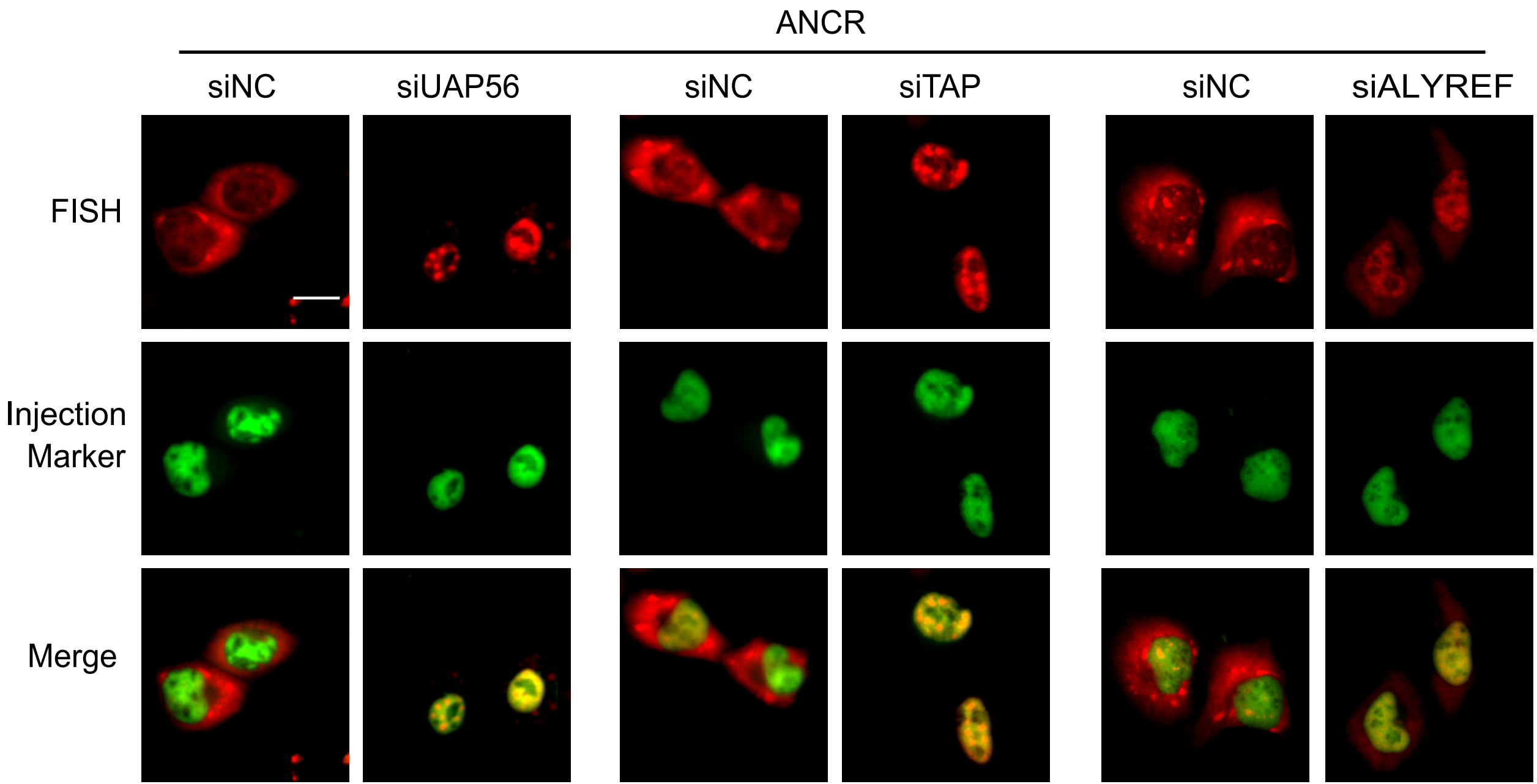
ANCR
siNC
siUAP56
siNC
siTAP
siNC
siALYREF
FISH
Injection Marker
Merge
