# Supplementary material for: Exon junction complex and nuclear pore complex components preferentially govern the nuclear export of spliced lncRNAs
Source: Cell Death Discov. 2026 Jul 8;12:392. doi: 10.1038/s41420-026-03246-0 (PMC13631367; doi:10.1038/s41420-026-03246-0)
Supplement: Supplementary file 9 [file 41420_2026_3246_MOESM4_ESM.pdf]

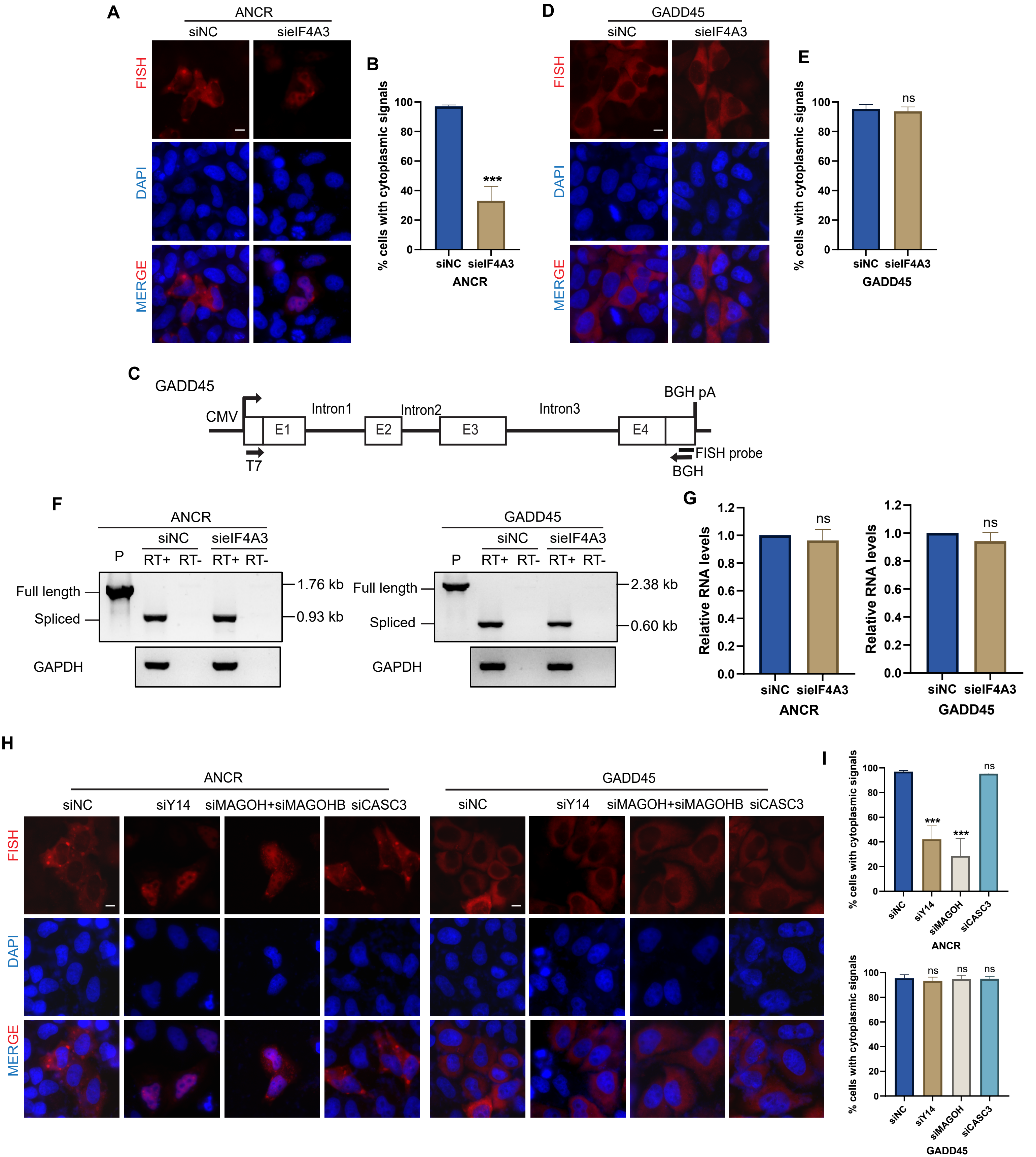

A
ANCR
siNC
sieIF4A3
FISH
DAPI
MERGE
B
% cells with cytoplasmic signals
100
80
60
40
20
0
***
siNC
sieIF4A3
ANCR
D
GADD45
siNC
sieIF4A3
FISH
DAPI
MERGE
E
% cells with cytoplasmic signals
ns
siNC
sieIF4A3
GADD45
C
GADD45
CMV
E1
Intron1
E2
Intron2
E3
Intron3
E4
BGH pA
T7
FISH probe
BGH
F
ANCR
siNC
sieIF4A3
P
RT+
RT-
RT+
RT-
Full length
Spliced
1.76 kb
0.93 kb
GAPDH
GADD45
siNC
sieIF4A3
P
RT+
RT-
RT+
RT-
Full length
Spliced
2.38 kb
0.60 kb
GAPDH
G
Relative RNA levels
1.2
1.0
0.8
0.6
0.4
0.2
0.0
ns
siNC
sieIF4A3
ANCR
Relative RNA levels
ns
siNC
sieIF4A3
GADD45
H
ANCR
siNC
siY14
siMAGOH+siMAGOHB
siCASC3
GADD45
siNC
siY14
siMAGOH+siMAGOHB
siCASC3
FISH
DAPI
MERGE
I
% cells with cytoplasmic signals
***
***
ns
siNC
siY14
siMAGOH
siCASC3
ANCR
% cells with cytoplasmic signals
ns
ns
ns
siNC
siY14
siMAGOH
siCASC3
GADD45
