# Supplementary material for: Exon junction complex and nuclear pore complex components preferentially govern the nuclear export of spliced lncRNAs
Source: Cell Death Discov. 2026 Jul 8;12:392. doi: 10.1038/s41420-026-03246-0 (PMC13631367; doi:10.1038/s41420-026-03246-0)
Supplement: Supplementary file 10 [file 41420_2026_3246_MOESM5_ESM.pdf]

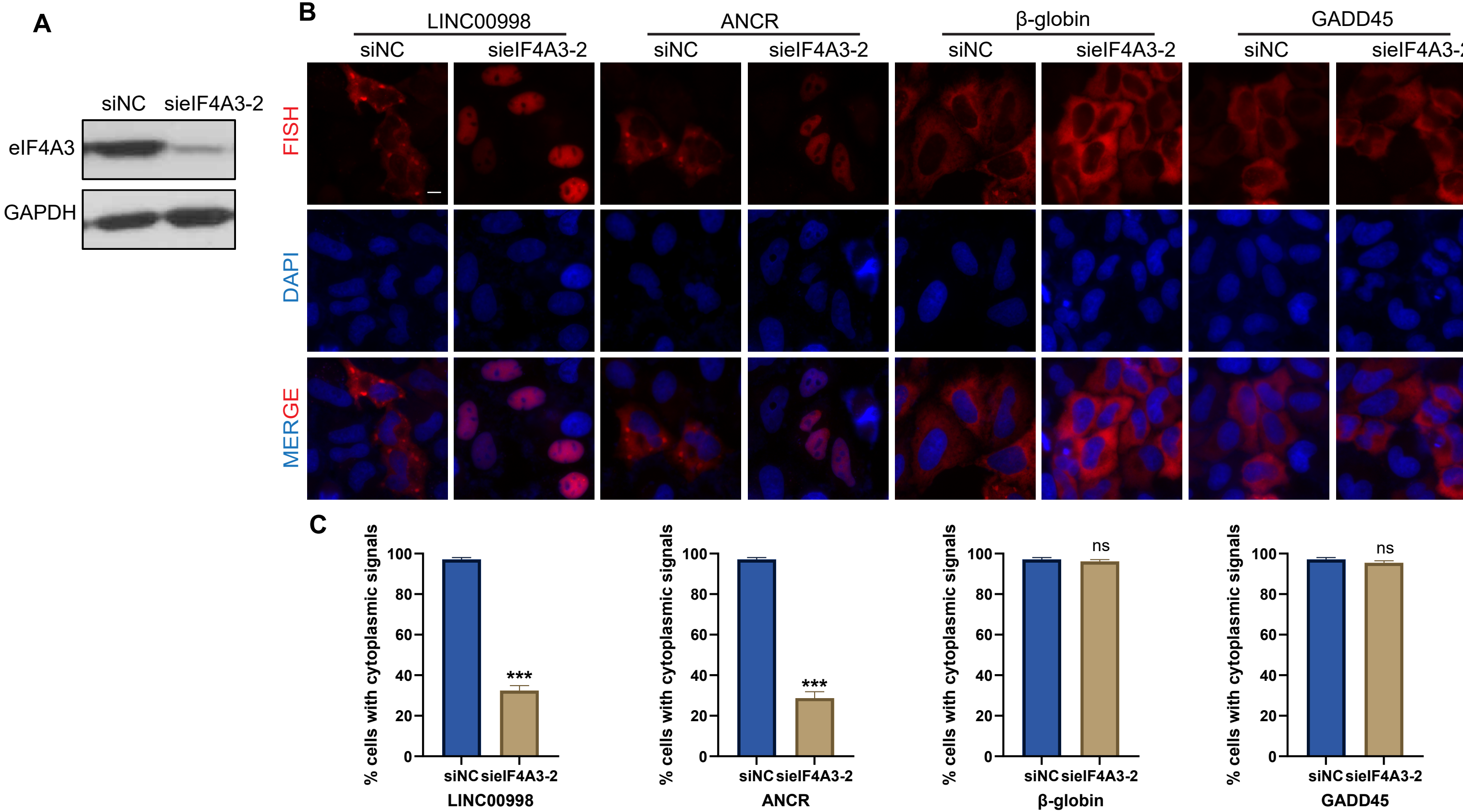
A
siNC
sieIF4A3-2
eIF4A3
GAPDH
B
LINC00998
ANCR
β-globin
GADD45
siNC
sieIF4A3-2
FISH
DAPI
MERGE
C
% cells with cytoplasmic signals
100
80
60
40
20
0
***
ns
LINC00998
ANCR
β-globin
GADD45
