# Supplementary material for: Exon junction complex and nuclear pore complex components preferentially govern the nuclear export of spliced lncRNAs
Source: Cell Death Discov. 2026 Jul 8;12:392. doi: 10.1038/s41420-026-03246-0 (PMC13631367; doi:10.1038/s41420-026-03246-0)
Supplement: Supplementary file 11 [file 41420_2026_3246_MOESM6_ESM.pdf]

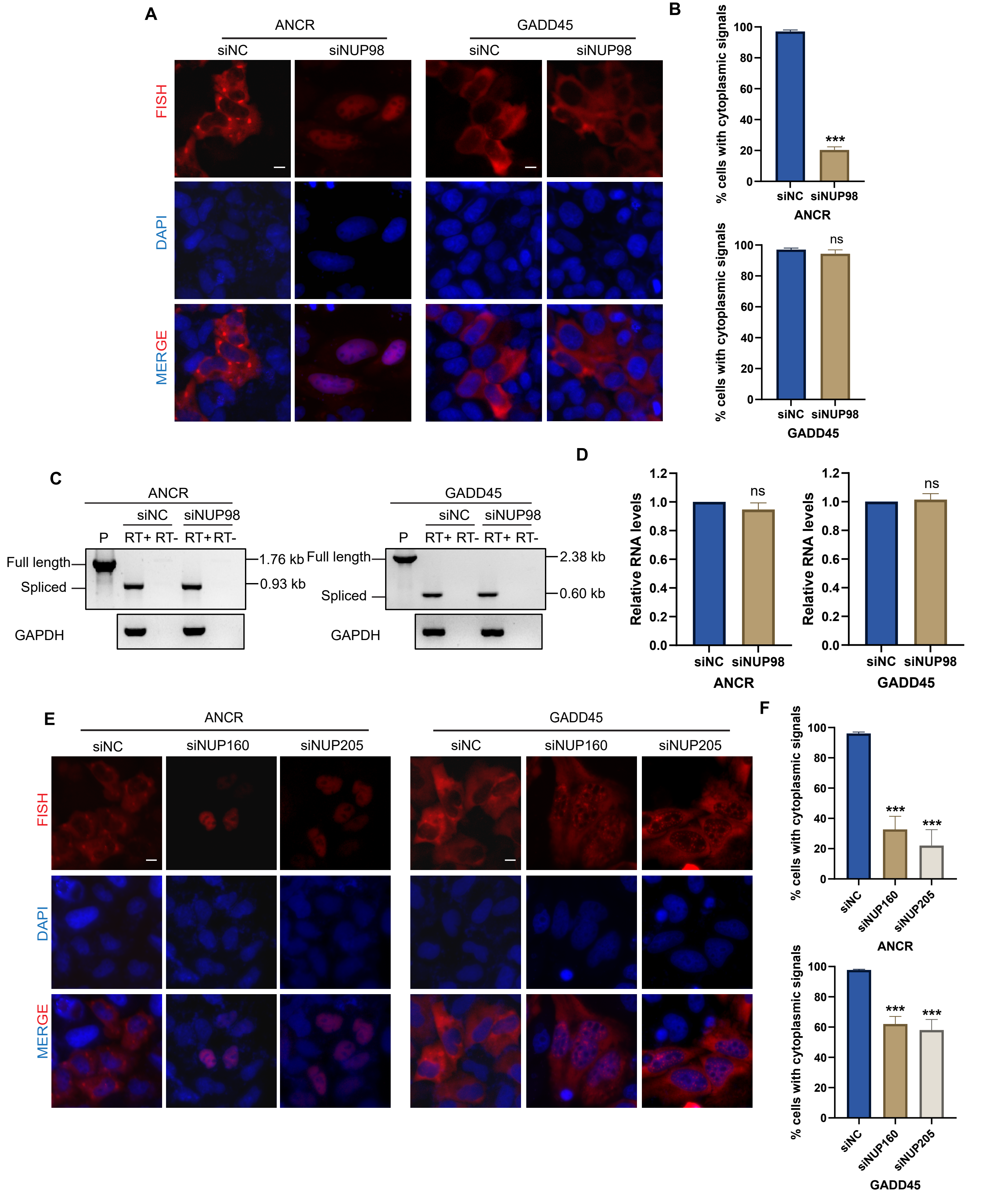
A
ANCR
GADD45
siNC
siNUP98
FISH
DAPI
MERGE
B
% cells with cytoplasmic signals
100
80
60
40
20
0
***
ns
siNC siNUP98
ANCR
GADD45
C
siNC siNUP98
P RT+ RT- RT+ RT-
Full length
Spliced
1.76 kb
0.93 kb
2.38 kb
0.60 kb
GAPDH
D
Relative RNA levels
1.2
1.0
0.8
0.6
0.4
0.2
0.0
E
siNUP160
siNUP205
F
siNC
siNUP160
siNUP205
