# Supplementary material for: Exon junction complex and nuclear pore complex components preferentially govern the nuclear export of spliced lncRNAs
Source: Cell Death Discov. 2026 Jul 8;12:392. doi: 10.1038/s41420-026-03246-0 (PMC13631367; doi:10.1038/s41420-026-03246-0)
Supplement: Supplementary file 13 [file 41420_2026_3246_MOESM8_ESM.pdf]

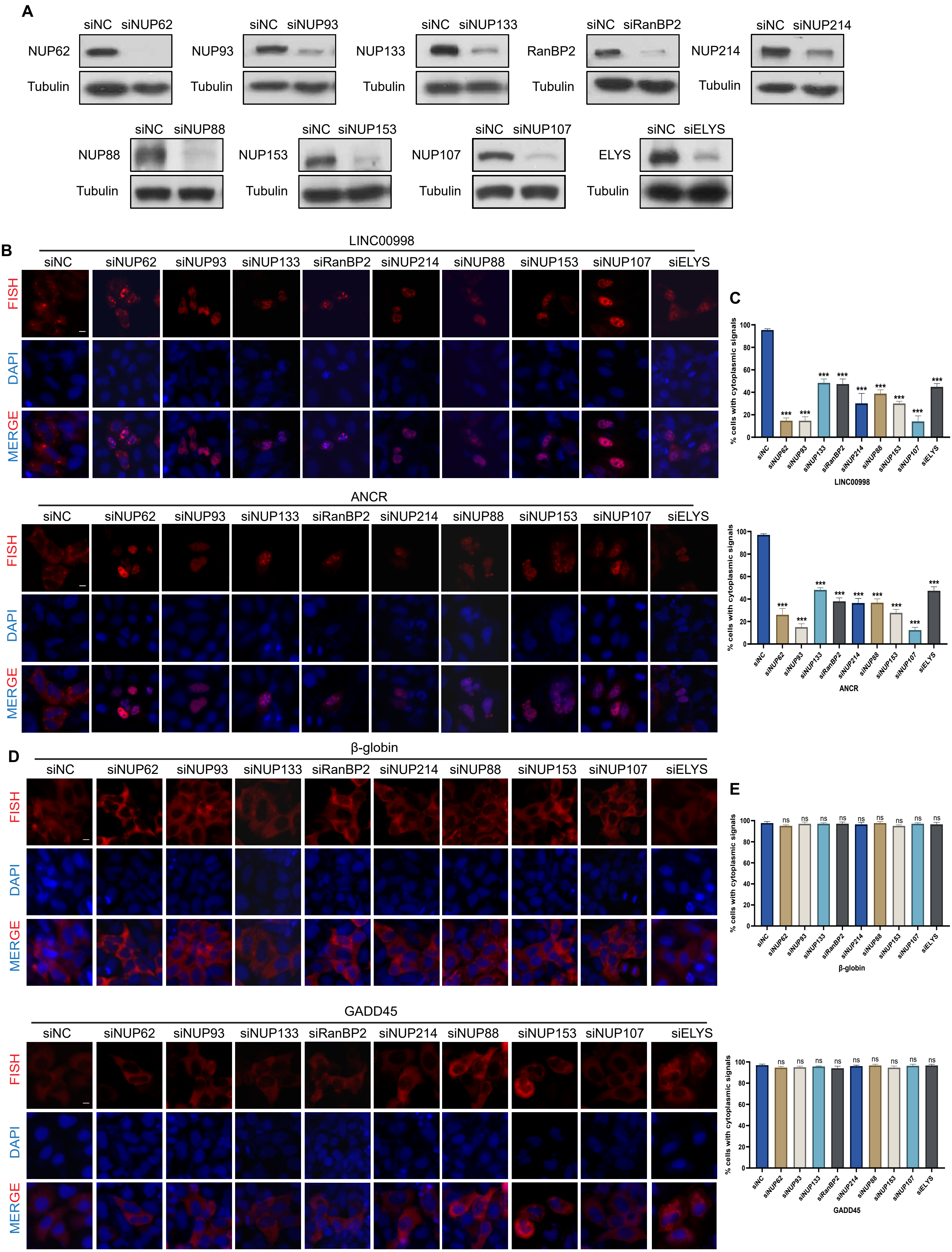
A
siNC siNUP62
NUP62
Tubulin
siNC siNUP93
NUP93
Tubulin
siNC siNUP133
NUP133
Tubulin
siNC siRanBP2
RanBP2
Tubulin
siNC siNUP214
NUP214
Tubulin
siNC siNUP88
NUP88
Tubulin
siNC siNUP153
NUP153
Tubulin
siNC siNUP107
NUP107
Tubulin
siNC siELYS
ELYS
Tubulin
B
LINC00998
siNC siNUP62 siNUP93 siNUP133 siRanBP2 siNUP214 siNUP88 siNUP153 siNUP107 siELYS
FISH
DAPI
MERGE
C
% cells with cytoplasmic signals
LINC00998
ANCR
siNC siNUP62 siNUP93 siNUP133 siRanBP2 siNUP214 siNUP88 siNUP153 siNUP107 siELYS
FISH
DAPI
MERGE
% cells with cytoplasmic signals
ANCR
D
β-globin
siNC siNUP62 siNUP93 siNUP133 siRanBP2 siNUP214 siNUP88 siNUP153 siNUP107 siELYS
FISH
DAPI
MERGE
E
% cells with cytoplasmic signals
β-globin
GADD45
siNC siNUP62 siNUP93 siNUP133 siRanBP2 siNUP214 siNUP88 siNUP153 siNUP107 siELYS
FISH
DAPI
MERGE
% cells with cytoplasmic signals
GADD45
