# Supplementary material for: Exon junction complex and nuclear pore complex components preferentially govern the nuclear export of spliced lncRNAs
Source: Cell Death Discov. 2026 Jul 8;12:392. doi: 10.1038/s41420-026-03246-0 (PMC13631367; doi:10.1038/s41420-026-03246-0)
Supplement: Supplementary file 14 [file 41420_2026_3246_MOESM9_ESM.pdf]

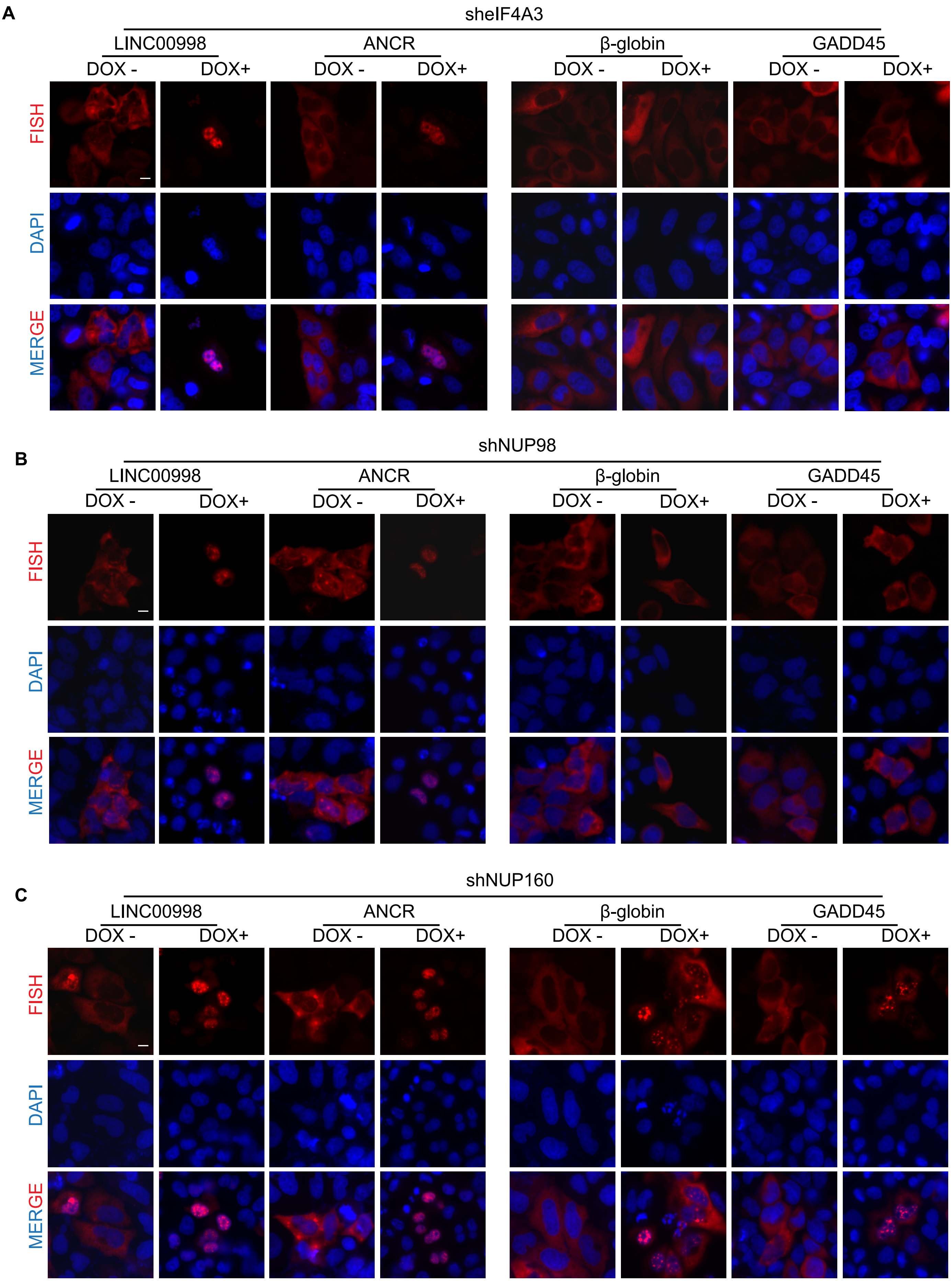
A
sheIF4A3
LINC00998
ANCR
β-globin
GADD45
DOX -
DOX+
FISH
DAPI
MERGE
B
shNUP98
C
shNUP160
