# Supplementary material for: Exon junction complex and nuclear pore complex components preferentially govern the nuclear export of spliced lncRNAs
Source: Cell Death Discov. 2026 Jul 8;12:392. doi: 10.1038/s41420-026-03246-0 (PMC13631367; doi:10.1038/s41420-026-03246-0)
Supplement: Supplementary file 15 [file 41420_2026_3246_MOESM1_ESM.docx]

**Supplementary figure legends**

**Supplementary Figure 1. Subcellular localization and lncRNPs purification of spliced lncRNA reporter ANCR**

A. **Schematic representation of the ANCR reporter construct in pcDNA 3.1, driven by the CMV promoter and terminated by the BGH poly(A) signal. White boxes represent exons, and black lines indicate introns.** T7 and BGH indicates plasmid specific primers.
B. **RNA-FISH analysis of nuclear and cytoplasmic distribution of ANCR reporter transcripts at the indicated time points following nuclear microinjection into HeLa cells. Transcription was blocked with α-amanitin 30 minutes after injection (defined as 0 hour).** Scale bar = 5 µm.
C. **RT-PCR analysis** using T7 and BGH primers, **confirming normal splicing of the ANCR reporter transcripts following microinjection.**
D. **Establishment of DOX-inducible FRT-HeLa cell lines stably expressing ANCR. Expression was induced by DOX treatment and detected by RT-PCR** using ANCR-KpnI-F and ANCR-ApaI-R primers. GAPDH is used as the loading control.
E. **Validation of nuclear and cytoplasmic fractionation by western blot using UAP56 and tubulin as nuclear and cytoplasmic markers, respectively.**
F. **Silver staining of purified ANCR lncRNPs from nuclear and cytoplasmic fractions showing distinct banding patterns.**
G. **Western blot analysis confirming the interaction of selected proteins identified by mass spectrometry with ANCR.**

**Supplementary Figure 2. TREX-TAP dependent export of lncRNA ANCR**

RNA-FISH analysis of nuclear and cytoplasmic localization of ANCR reporter transcripts post-microinjection following knockdown of UAP56, TAP, or ALYREF. Scale bar = 5 µm.

**Supplementary Figure 3. eIF4A3 and other core EJC components selectively export of spliced lncRNA ANCR but not mRNA GADD45**

A. Subcellular localization of ANCR reporter transcripts post-transfection following eIF4A3 knockdown. Scale bar = 10 µm.
B. Quantification of cells with predominant cytoplasmic signals for ANCR following eIF4A3 knockdown (n=3, ***: p < 0.001).
C. Schematic representation of the spliced GADD45 mRNA reporter **construct in pcDNA 3.1, driven by the CMV promoter and terminated by the BGH poly(A) signal. White boxes represent exons, and black lines indicate introns.** T7 and BGH indicates plasmid specific primers.
D. Subcellular localization of GADD45 reporter transcripts post-transfection following eIF4A3 knockdown. Scale bar = 10 µm.
E. Quantification of cells with predominant cytoplasmic signals for GADD45 following eIF4A3 knockdown (n=3, ns: not significant).
F. RT-PCR analysis of splicing and overall abundance of ANCR and GADD45 reporter transcripts following eIF4A3 knockdown using T7 and BGH primers. P: plasmid control; RT+: reverse transcriptase added; RT-: no reverse transcriptase control
G. Quantification of relative RNA levels of ANCR and GADD45 following eIF4A3 knockdown showing no significant change in RNA abundance (n=3, ns: not significant).
   H. RNA-FISH analysis of subcellular localization of ANCR and GADD45 reporter transcripts following knockdown of Y14, MAGOH/MAGOHB, and CASC3. Scale bar = 10 µm.
   I. Quantification of cells with predominant cytoplasmic signals for ANCR and GADD45 following knockdown of individual EJC components (n=3, ns: not significant, ***: p < 0.001).

**Supplementary Figure 4. A second independent siRNA targeting eIF4A3 confirms lncRNA-biased nuclear retention.**

A. **Western blot validation of siRNA-mediated knockdown of eIF4A3 using a second independent siRNA (sieIF4A3-2) in HeLa cells. GAPDH serves as a loading control.**
B. **RNA-FISH analysis of subcellular localization of LINC00998, ANCR, β-globin, and GADD45 reporter transcripts following sieIF4A3-2 knockdown. Scale bar = 10 µm.**
C. **Quantification of cells with** predominant **cytoplasmic signals for LINC00998, ANCR, β-globin, and GADD45 following sieIF4A3-2 knockdown. (n=3, ns: not significant, ***: p < 0.001).**

**Supplementary Figure 5. Validation of RNA reporters’ localization under NUP98, NUP160, and NUP205 depletion.**

A. RNA-FISH analysis of subcellular localization of ANCR and GADD45 reporter transcripts post-transfection following NUP98 knockdown. Scale bar = 10 µm.

B. Quantification of cells with predominant cytoplasmic signals for ANCR and GADD45 following NUP98 knockdown (n=3, ns: not significant, ***: p < 0.001).
C. RT-PCR analysis of splicing and overall abundance of ANCR and GADD45 reporter transcripts following NUP98 knockdown using T7 and BGH primers. P: plasmid control; RT+: reverse transcriptase added; RT-: no reverse transcriptase control.
D. Quantification of relative RNA levels of ANCR and GADD45 following NUP98 knockdown showing no significant change in RNA abundance (n=3, ns: not significant).
E. RNA-FISH analysis of subcellular localization of ANCR and GADD45 reporter transcripts following knockdown of NUP160 and NUP205 Scale bar = 10 µm.
F. Quantification of cells with predominant cytoplasmic signals for ANCR and GADD45 following knockdown of NUP160 and NUP205 (n=3, ***: p < 0.001).

**Supplementary Figure 6. A second independent siRNA targeting NUP98 confirms lncRNA-biased nuclear retention.**

A. Western blot validation of siRNA-mediated knockdown of NUP98 using a second independent siRNA (siNUP98-2) in HeLa cells. Tubulin serves as a loading control.
B. RNA-FISH analysis of subcellular localization of LINC00998, ANCR, β-globin, and GADD45 reporter transcripts following siNUP98-2 knockdown. Scale bar = 10 µm.
C. Quantification of cells with predominant cytoplasmic signals for LINC00998, ANCR, β-globin, and GADD45 following siNUP98-2 knockdown. (n=3, ns: not significant, ***: p < 0.001).

**Supplementary Figure 7. A subset of NPC components orchestrates the biased nuclear export of spliced lncRNAs**

A. Western blot validation of siRNA-mediated knockdown of NUPs in HeLa cells.

B. RNA-FISH analysis showing nuclear retention of lncRNA reporter transcripts post-transfection following depletion of selected NUPs. Scale bar = 10 µm.
C. Quantification of cells with predominant cytoplasmic signals for lncRNA reporter transcripts following depletion of selected NUPs. (n=3, ***: p < 0.001).
D. RNA-FISH analysis showing subcellular localization of mRNA reporter transcripts post-transfection following depletion of selected NUPs. Scale bar = 10 µm.
E. Quantification of cells with predominant cytoplasmic signals for mRNA reporter transcripts following depletion of selected NUPs. (n=3, ns: not significant).

**Supplementary Figure 8. Validation of RNA reporters’ localization in DOX-inducible knockdown stable cell lines of eIF4A3, NUP98, and NUP160**

A-C. RNA-FISH showing the localization of lncRNA reporters (LINC00998, ANCR) and mRNA reporters (β-globin, GADD45) transcripts. Scale bar = 10 µm.

**Supplementary Figure 9. eIF4A3 knockdown does not affect splicing efficiency of endogenous lncRNA**

A. Schematic representation of KRT10-AS1-201 exon-intron organization showing the location of exon-exon (EE) and exon-intron (EI) primer pairs used to detect spliced and unspliced transcripts respectively.
B. RT-PCR analysis of endogenous spliced and unspliced KRT10-AS1-201 transcripts using EE and EI primer pairs in control (DOX-) and eIF4A3 knockdown (DOX+) whole cells. RT+: reverse transcriptase added; RT-: no reverse transcriptase control.
C. Quantification showing no significant change in splicing efficiency of endogenous lncRNA KRT10-AS1-201 upon eIF4A3 knockdown (n=3, ns: not significant).

**Supplementary Figure 10. Correlation of GC content with nuclear retention.**

Scatter plots showing the correlation between GC content (%) and log₂(sh knockdown_NTR/shNC_NTR) (shknockdown = DOX+, shNC = DOX-) for lncRNAs and mRNAs following eIF4A3 (A, B), NUP98 (C, D), and NUP160 (E, F) knockdown. The nuclear-to-total RNA ratio (NTR) was calculated as NTR = FPKM_NE / (FPKM_NE + FPKM_CE). Spearman correlation coefficients and p-values are indicated. Red line represents the linear regression fit.

**Supplementary Figure 11 Correlation of exon number with nuclear retention.**

Scatter plots showing the correlation between exon number and log₂(shknockdown_NTR/shNC_NTR) (shknockdown = DOX+, shNC = DOX-) for lncRNAs and mRNAs following eIF4A3 (A, B), NUP98 (C, D), and NUP160 (E, F) knockdown. The nuclear-to-total RNA ratio (NTR) was calculated as NTR = FPKM_NE / (FPKM_NE + FPKM_CE). Spearman correlation coefficients and p-values are indicated. Red line represents the linear regression fit.

**Supplementary Figure 12. Correlation of transcript length with nuclear retention.**

Scatter plots showing the correlation between transcript length and log₂(shknockdown_NTR/shNC_NTR) (shknockdown = DOX+, shNC = DOX-) for lncRNAs and mRNAs following eIF4A3 (A, B), NUP98 (C, D), and NUP160 (E, F) knockdown. The nuclear-to-total RNA ratio (NTR) was calculated as NTR = FPKM_NE / (FPKM_NE + FPKM_CE). Spearman correlation coefficients and p-values are indicated. Red line represents the linear regression fit.

**Supplementary Figure 13. Correlation of splice site strength with nuclear retention.**

Scatter plots showing the correlation between median 5' splice site score (median_5ss_score) and median 3' splice site score (median_3ss_score) with log₂(shknockdown_NTR/shNC_NTR) (shknockdown = DOX+, shNC = DOX-) for lncRNAs and mRNAs following eIF4A3 (A–D), NUP98 (E–H), and NUP160 (I–L) knockdown. The nuclear-to-total RNA ratio (NTR) was calculated as NTR = FPKM_NE / (FPKM_NE + FPKM_CE). Spearman correlation coefficients and p-values are indicated. Red line represents the linear regression fit.
